# Robust tetra‐armed poly (ethylene glycol)‐based hydrogel as tissue bioadhesive for the efficient repair of meniscus tears

**DOI:** 10.1002/mco2.738

**Published:** 2024-10-24

**Authors:** Jing Ye, Yourong Chen, Ronghui Deng, Jiying Zhang, Hufei Wang, Shitang Song, Xinjie Wang, Bingbing Xu, Xing Wang, Jia‐Kuo Yu

**Affiliations:** ^1^ Sports Medicine Department Beijing Key Laboratory of Sports Injuries Peking University Third Hospital Beijing Haidian District China; ^2^ Institute of Sports Medicine Peking University Beijing Haidian District China; ^3^ Beijing National Laboratory for Molecular Sciences Institute of Chemistry Chinese Academy of Sciences Beijing China; ^4^ University of Chinese Academy of Sciences Beijing China; ^5^ Orthopaedic and Sports Medicine Center Beijing Tsinghua Changgung Hospital Tsinghua University Beijing China; ^6^ Institute of Orthopedic and Sports Medicine of Tsinghua Medicine Tsinghua University Beijing China

**Keywords:** bioadhesive, longitudinal tear, meniscus, polyethylene glycol, tissue adhesives

## Abstract

Repair and preservation of the injured meniscus has become paramount in clinical practice. However, the complexities of various clinic stitching techniques for meniscus repair pose challenges for grassroots doctors. Hence, there is a compelling interest in innovative therapeutic strategies such as bioadhesives. An ideal bioadhesive must cure quickly in aqueous and blood environments, bind strongly, endure arthroscopic washing pressures, and degrade appropriately for tissue regeneration. Here, we present a tetra‐poly (ethylene glycol) (PEG)‐based hydrogel bioadhesive, boasting high biocompatibility, ultrafast gelation, facile injectable operation, and favorable mechanical strength. In view of the synergistic effects of chemical anchor and physical chain entanglement to tightly bind the meniscus, a seamless interface was formed between the surrounding meniscal tissues and hydrogels, enabling the longitudinal tear of the meniscus fused in situ to withstand large tensile force with the adhesive strength of 541.5 ± 31.4 kPa and arthroscopic washout resistance of 29.4 kPa. Superior to existing commercial adhesives, ours allows sutureless application and arthroscopic assistance, without requiring specialized clinical skills. This research is expected to significantly impact our understanding of meniscal healing and ultimately promote a simpler process for achieving functional and structural recovery in torn menisci.

## Graphical abstract

Tetra‐PEG hydrogel bioadhesive schematic diagram for meniscal repair. More specifically the key points of our study are:

1. Facile construction of the applicable tetra‐PEG bioadhesive. Once synchronously sprayed onto the injured meniscus tissues, NHS‐active tetra‐PEG‐SC solution can simultaneously be reacted with amine groups of tetra‐PEG‐NH_2_ solution and tissue proteins of the cartilage surfaces within seconds, contributing to the high mechanical toughness and long‐term tissue adherence.

2. Of particular collaborative mechanism of strengthened hydrogel‒meniscus adhesion. In views of the synergistic effects of robust chemical anchor between the amine‐abundant proteins and NHS‐active ester hydrogels and strong physical entanglements between the meniscus fibers and polymeric chains, this bioadhesive can support higher interfacial toughness by dissipating a substantial amount of mechanical energy when there are outside forces after clinical application.

3. A progressive option for the clinical complex meniscus injury. Without tedious manipulation procedure and specialized clinical skills, this novel bioadhesive can be tightly integrated with the surrounding cartilage tissue to withstand a hydraulic pressure of 29.4 kPa when flushed with water, far exceeding the clinically arthroscopic surgery (∼10 kPa) and existing commercial adhesives, which can be clinically applied to promote meniscus healing and chondroprotection.

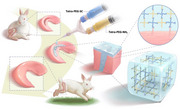

## 1 INTRODUCTION

The meniscus, a wedge‐shaped between the femoral condyle and the tibia plateau, can tolerate a variety of stresses coming from all angles, including compression, tension, rip, etc. One of the most serious musculoskeletal morbidities, meniscal damage can limit knee function, cause excruciating pain, and possibly lead to disability.^1^, ^2^ Presently, meniscus injuries are increasingly prevalent due to an aging population and rising sports‐related accidents. Each year, over 1.7 million individuals worldwide require meniscus surgery.^3^ Although conventional meniscectomy strategy can partially relieve the pain, there is still a chance of osteoarthritis. The meniscal suture has been a widespread treatment option for meniscus injuries, along with the advancement of arthroscopic procedures and the spread of the therapeutic idea of maintaining meniscus function.^4^ However, numerous technical challenges persist in meniscus suture repair, particularly with all‐inside meniscal sutures. These issues stem from insufficient surgical expertise, limited operational visibility, and inexperienced assistance, leading to inappropriate suture techniques, iatrogenic cartilage injury, and anchor loss.^5^, ^6^, ^7^ To tackle these challenges, innovative therapeutic methods and simple surgical means for clinically complex meniscus injuries are highly demanded.

Bioadhesives, viewed as a promising alternative for wound closure, have the potential to revolutionize medical procedures due to their non‐invasive nature and ease of application without specialized requirements. However, current tissue bioadhesives approved for medical use, such as natural fibrin glues (TISSEEL) and cross‐linked polyethylene glycols (COSEAL), present significant limitations including inadequate mechanical strength on tissue surfaces, potential risks of viral and disease transmission, and allergic reactions.^8^, ^9^, ^10^, ^11^ Moreover, commercially available cyanoacrylates such as Dermabond are synthetic tissue adhesives known for their high strength. However, their applications in the human body are greatly restricted due to factors such as exothermal polymerization, prolonged retention time, and the potential presence of toxic degradation products.^12^, ^13^ Although a few blood‐resistant tissue adhesives with better adhesion performance have been developed, their potential for use in clinical settings is severely limited by the need for ultraviolet (UV) irradiation^14^, ^15^, ^16^ and/or the continuous application of constant pressure.^17^, ^18^, ^19^ However, the majority of hydrogel bioadhesives still struggle to maintain stability and cohesion within the clinically complex meniscus, particularly in the challenging environment created by standard arthroscopic procedures. These procedures involve fluid irrigation during surgery to ensure clear vision, necessitating constant fluid irrigation under a hydraulic pressure of 10 kPa, which can compromise the effectiveness of the adhesive.^20^ Particularly, the applied tissue bioadhesive for the clinical complex meniscus injury should be able to operate easily for novice clinicians, gelatinize and solidify quickly, firmly adhere to the tissue around them and keep up a high level of mechanical stability to endure pressure from the environment.^21^, ^22^

**SCHEME 1:**
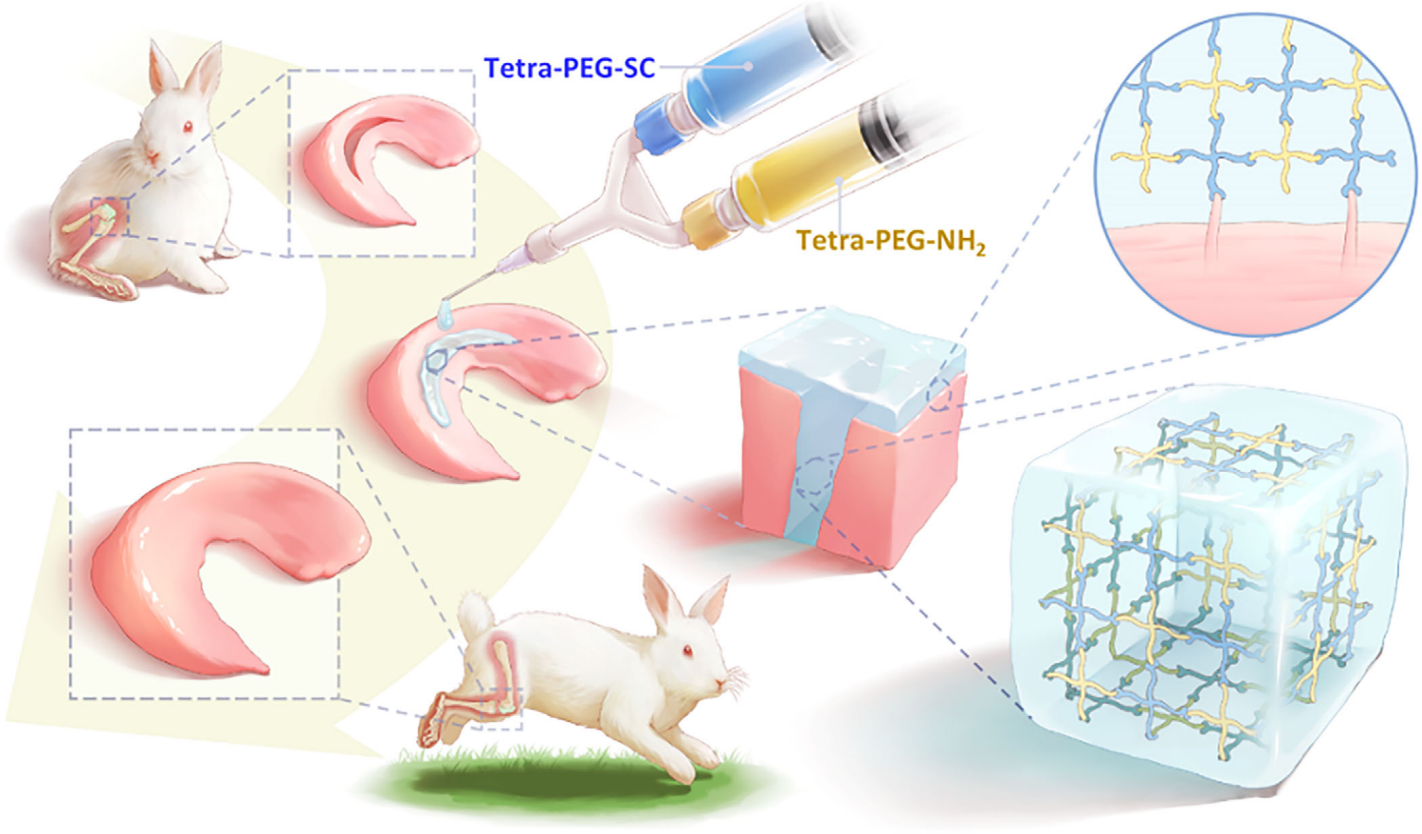
Tetra‐poly (ethylene glycol) (PEG) hydrogel bioadhesive schematic diagram for meniscal repair.

We developed a tetra‐poly (ethylene glycol) (PEG) hydrogel bioadhesive for sutureless sealing, which quickly binds to tissue proteins or cartilage, ensuring firm meniscus adhesion and pressure resistance. Its mechanical toughness supports tissue dynamics, promoting cell development, RNA transcription, proliferation, migration, and regeneration. The bioadhesive's rapid gelation, strength, adhesion, and injectability enable efficient meniscus repair in vitro and in vivo. In addition to good tissue anchorage and compliance to wet meniscus and efficient meniscus repair and chondroprotection (Scheme 1).

## 2 RESULTS

### 2.1 Preparation and characterization of the tetra‐PEG hydrogel adhesive

**FIGURE 1:**
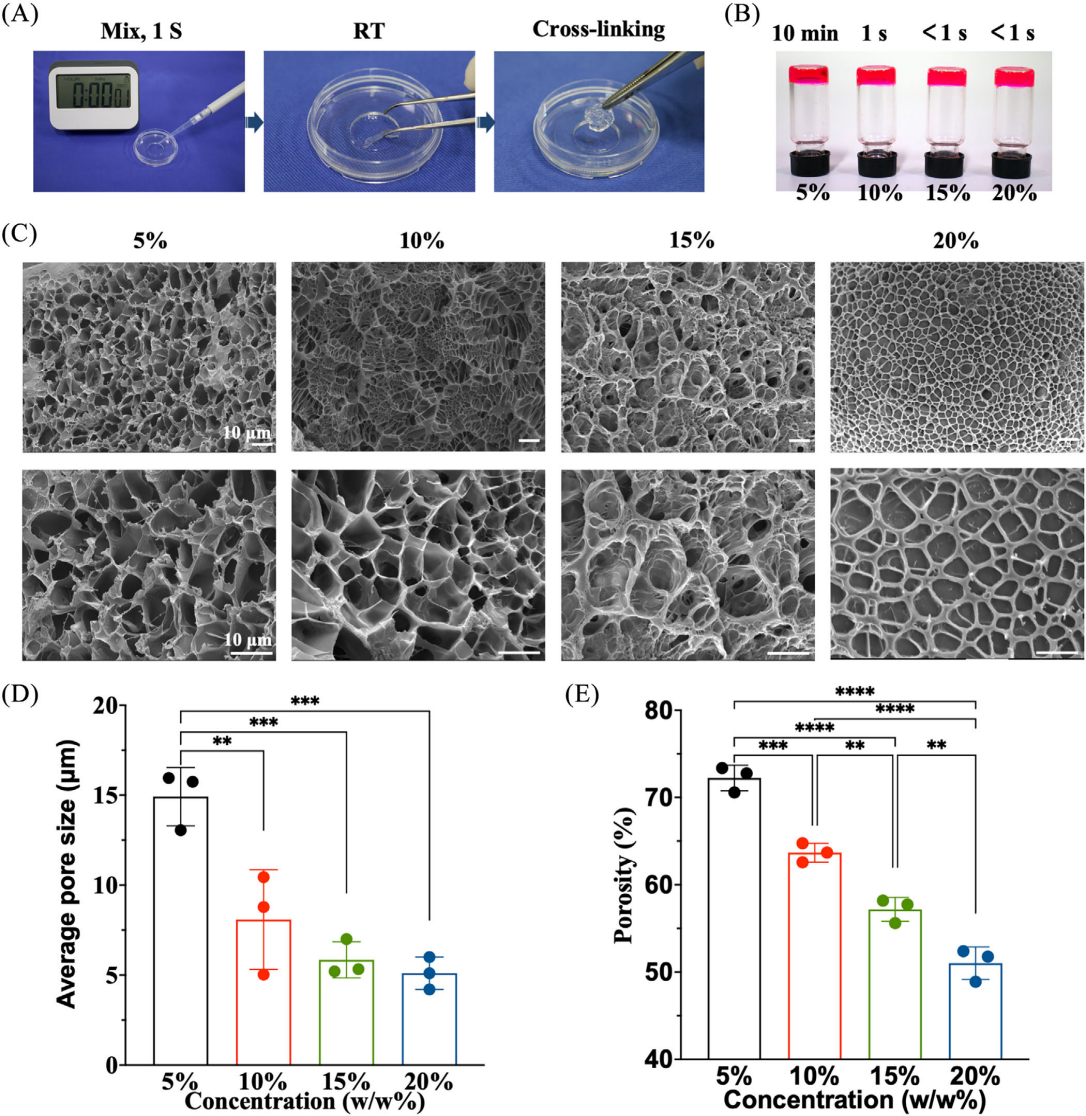
Ultrafast gelation and microstructures of tetra‐poly (ethylene glycol) (PEG) hydrogel with various solid contents. (A) Photographs showing rapid gelation of the tetra‐PEG hydrogel. (B) Gelation time; (C) scanning electron microscopy (SEM) images; (D) average pore size; (E) porosity; (F) water retention profile of tetra‐PEG hydrogel with varied solid contents at 5%, 10%, 15%, and 20%.

Tetra‐PEG‐SC and tetra‐arm PEG‐amine (tetra‐PEG‐NH_2_) polymers were totally synthesized, avoiding any concerns that they would be hindered by anticoagulants or transferring disease (Figures S1 and S2). After mixing the tetra‐PEG‐NH_2_ and tetra‐PEG‐SC solutions in a dual syringe, an injectable tetra‐PEG hydrogel was promptly created within 1 s thanks to a very efficient ammonolysis reaction between the amine and active ester groups (Figures 1A,B and S3). High adhesion strength to the tissue was simultaneously produced during this gelling phase by forming a chemical bond between the amine groups of the tissue's proteins and the N‐hydroxysuccinimide (NHS)‐activated tetra‐PEG‐SC polymer. After manipulation of the solid content, gelatin time, porous structures, average pore size, and porosity as well as the water retention properties were easily tailored for facilitating the exchange of nutrients and cell growth (Figure 1C‒E and S22; Table S1).

We conducted strain amplitude sweep analysis, X‐ray diffraction analyses, fourier fransform infrared spectrometer (FTIR) analyses, thermogravimetric Analysis (TGA) analyses, thermal stability analysis of biological macromolecules (DSC) analyses, and Cell Counting Kit‐8(CCK‐8) cell proliferation experiments on the bioadhesive (Figures S4‒S11). Results indicate that bioadhesives with 5%, 10%, 15%, and 20% solid content exhibit comparable mechanical compression resistance and cytocompatibility, as well as adhesion capabilities. However, the bioadhesive with 5% solid content exhibited excessively long gelation time, exceeding clinical application requirements, and was therefore excluded. Conversely, the bioadhesives with 15% and 20% solid content exhibited gelation times of less than 1 s, making them impractical for application as they solidify too quickly for subsequent extrusion to the desired bonding site. Taking consideration of the least economic costs and stable microstructure, 10% of solid content was selected as a suitable usage concentration for detailed investigation.

### 2.2 Mechanical, adhesion performance, and cytocompatibility of tetra‐PEG bioadhesive

**FIGURE 2:**
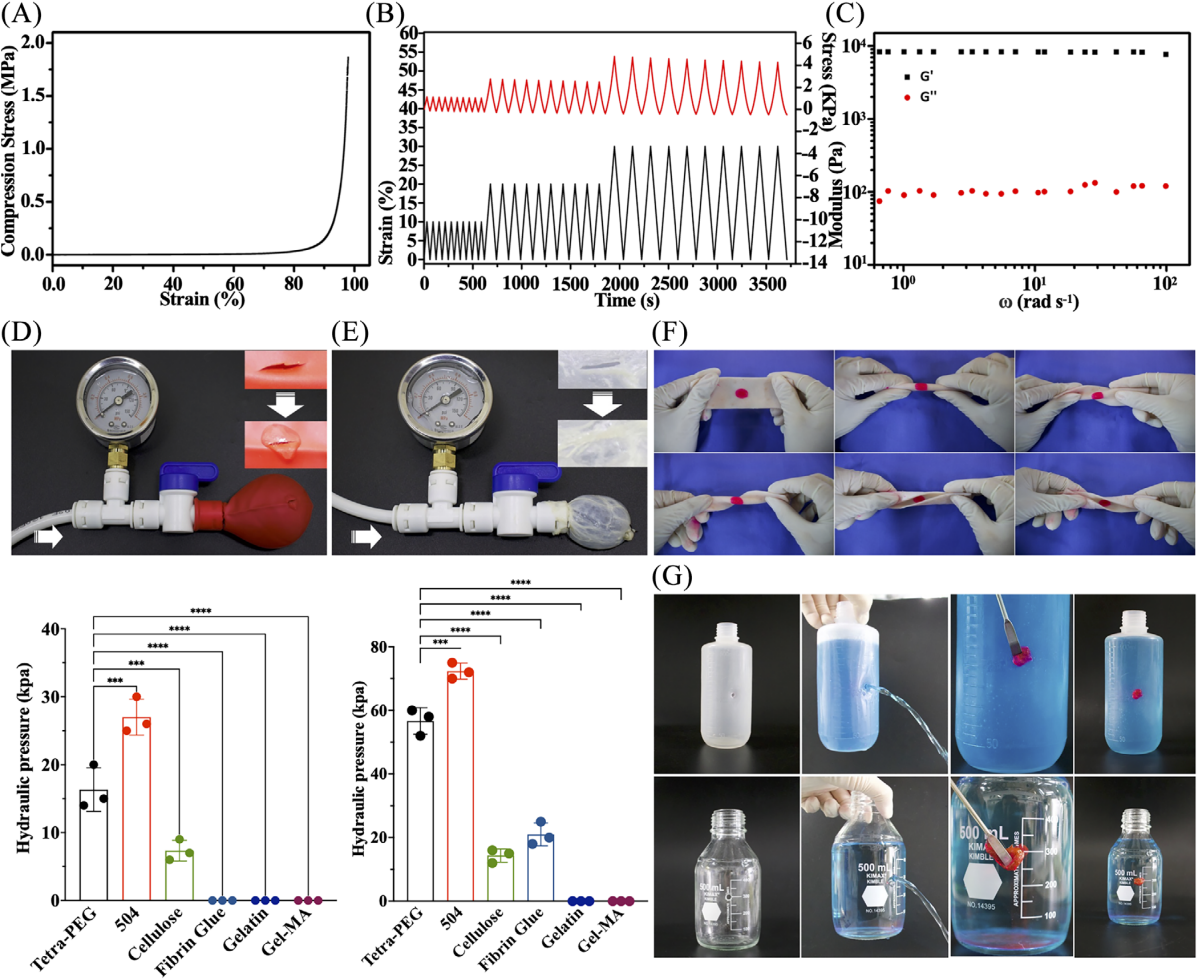
Mechanical and adhesion behavior of tetra‐poly (ethylene glycol) (PEG) bioadhesive. (A) Compression stress and (B) rheological analysis of the tetra‐PEG hydrogel (the black lines refer to strain; the red lines refer to stress). (C) The hydrogel was compressed 10 times (10 times at each strain): 10%, 20%, and 30%. (D and E) The binding strength is evaluated using a balloon and porcine small intestine burst pressure tests. (F) Adhesive strength via multi‐angle folding on the flat porcine skin. (G) Bonding and blocking assessment using PE and glass bottle breakage models.

As soon as the hydrogels came into contact with the tissues, the remaining NHS‐active ester in the hydrogel network interacted with amino groups on tissue proteins to create solid chemical connections and uniform network architectures between the hydrogels and tissues. In contrast to marketed cellulose, fibrin glue, and gelatin, these chemical linkages enabled a tight connection between tissues and the hydrogels with greater compression (Figure 2A), assuring that the hydrogels will stay stable even when there are external pressures after application. Furthermore, the mechanical strength of tetra‐PEG hydrogels did not noticeably decrease during the compression cycle, and repeated compression tests at 10%, 20%, and 30% strains (Figure 2B). The rheological result further demonstrated the tetra‐PEG hydrogel's ability to retain stability in a wide frequency range (Figure 2C) in a single step even in a damp environment within the knee articular cavity.

We initially use the balloon and pig small intestine (thickness = 0.15 mm) as a model for bursting pressure testing to evaluate the adhesion performance. An incision of 0.5 cm was made in the ruptured tissue model using a surgical knife, and it was then sealed with tetra‐PEG bioadhesive and another commercial adhesive by gently pushing for 30 s. The hydraulic pressure imitating blood flow was used to assess the bursting pressure after the lacerated balloon and pig small intestine were securely closed after 30 min. The pig small intestine and the sealed balloon could withstand hydraulic pressures of 14 and 60 kPa (Figures 2D,E and S12) using the tetra‐PEG bioadhesive, significantly over the 10 kPa restriction necessary for arthroscopic treatment. Note that although the α‐butyl cyanoacrylate 504 exhibited the best blocking effect, its potential toxicity hindered the wide applications for tissue adhesion and wound closure in the clinic.^21^ Additionally, the bioadhesive could be firmly bonded into the curled and folded pork despite various torsion angles (fold 180°, twist ±180°) (Figure 2F), and its satisfactory adhesion and sealing effect on the leaky PE bottle (hole: 7.5 cm above bottle bottom) and glass bottle (hole: 6 cm above bottle bottom) was also verified, with no exudation around the gels (Figure 2G).

To further prove the sealing ability in the clinic, a series of the extremely classical hemostatic model were performed to observe the wound healing of injured rabbit organs. The rapid and efficient hemostasis of damaged organs (skin, heart, liver, spleen, and blood vessels) forcefully demonstrated the remarkable sealing effect and adhesion performance of tetra‐PEG bioadhesive in vivo, laying a solid foundation for the clinical applications (Figure S13). As far as we know, PEG is a non‐toxic, FDA‐approved biomaterial that is frequently employed in engineering scaffold applications.

### 2.3 In vitro meniscus adhesion and bonding mechanism of tetra‐PEG bioadhesive

**FIGURE 3:**
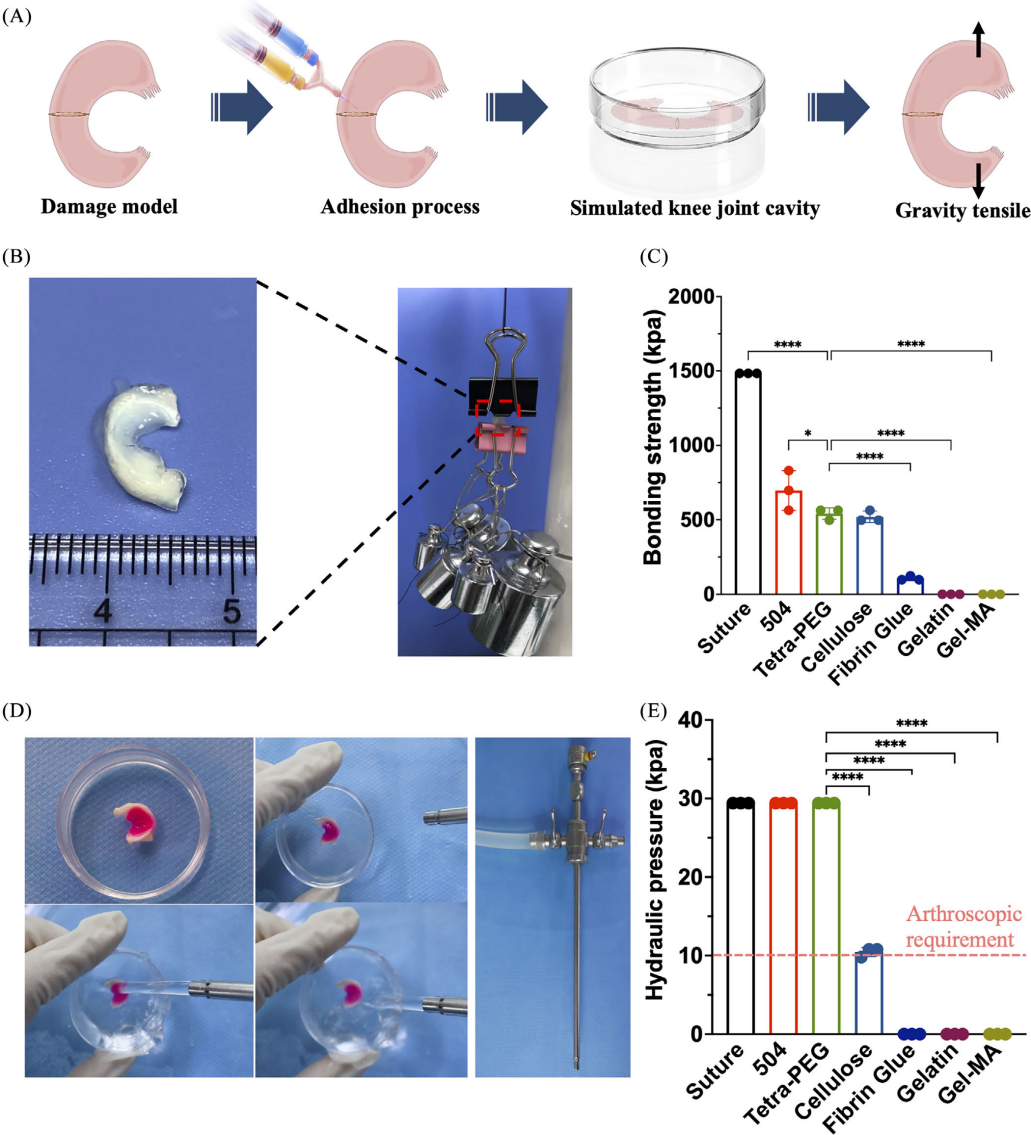
Evaluation of tetra‐poly (ethylene glycol) (PEG) bioadhesive for meniscal repair in vitro. (A and B) The gravity tensile testing procedure using the tetra‐PEG bioadhesive. (C) Quantitative of the gravity tensile test using various commercialized adhesives and tetra‐PEG bioadhesive. (D) Using an arthroscopy instrument, the cured tetra‐PEG bioadhesive–meniscal constructions demonstrated resistance to water pressure. (E) The meniscal defect repair's quantitative resistance to hydraulic pressure when washed with a variety of commercial adhesives and tetra‐PEG bioadhesives (group *n* = 3, ^****^ *p*<0.001).

To verify the possibility of gel sticking to the meniscus, we designed an in vitro model of meniscal injury to assess the adhesion effects of surgical sutures, commercialized approved adhesives for medical use and tetra‐PEG bioadhesive on the broken ends of the meniscus (Figure 3A). After placing in a closed phosphate buffer saline (PBS) petri dish to simulate the moist state in the knee cavity, the repaired meniscus was stretched by weight testing experiments. Figure 3B,C shows that the surgical suture group had the strongest tension, followed by 504, tetra‐PEG bioadhesive (541.5 ± 31.4 kPa) and cellulose. Notably, tetra‐PEG bioadhesive is preferred for underwater arthroscopic therapy based on its excellent cytocompatibility, strong mechanical strength, quick gelation, and good tissue attachment. The need for fluid irrigation during arthroscopic surgery necessitates the employment of quick‐switch injection instruments to stop the hydrogel precursor from diffusing when immersed in water and away from the intended area (it needs to solidify before being exposed to water). Tetra‐PEG bioadhesive was strongly integrated with the meniscus tissue around it, as seen in Figure 3D, and was able to tolerate a hydraulic pressure of 29.4 kPa when water is thrown at it. The pressure is far higher than the 10 kPa that is generally needed for arthroscopic therapy. In comparison, other commercialized adhesives, due to the slow gelation or weak adhesion, were easily washed away and cannot withstand this hydraulic pressure (Figure 3E), in which it was shown that the tetra‐PEG hydrogel, which may be used directly and steadily in meniscus bonding, performs better than other materials (consistent hydrostatic irrigation is crucial to achieve optimal visualization during the surgery).

### 2.4 In vitro meniscus adhesion and bonding physical mechanism of tetra‐PEG bioadhesive

**FIGURE 4:**
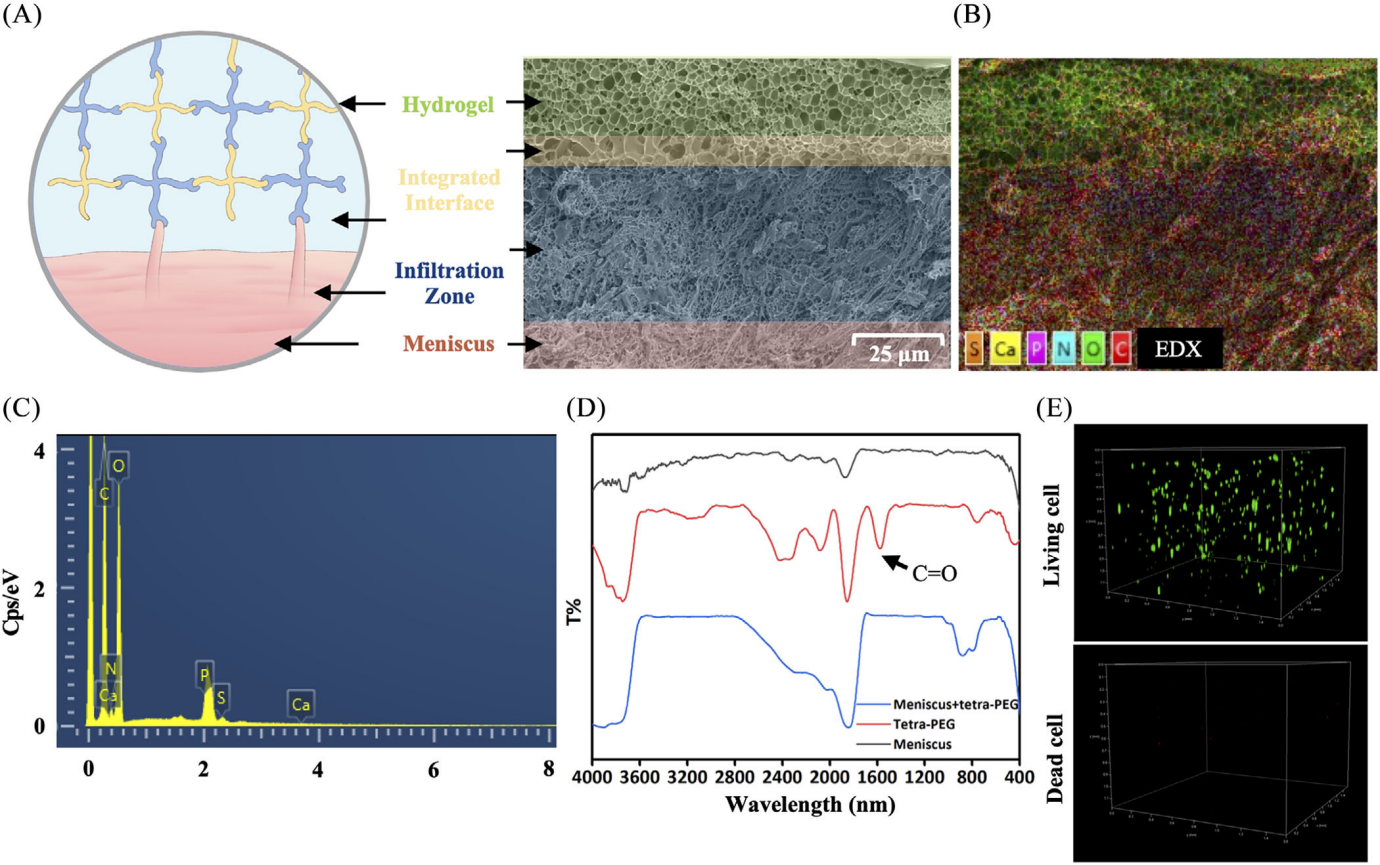
Tetra‐poly (ethylene glycol) (PEG) hydrogel–meniscal construct integrated interface. (A) Chemical bonding at the interface structure is depicted schematically, and the associated scanning electron microscopy (SEM) picture shows the hydrogel–meniscal complex. (B) EDX analysis of the interface structure of the hydrogel–meniscal construct. (C) Specific C, N, O, P, S, Ca analysis at the interface structure of hydrogel–meniscal construct. (D) FTIR spectra of untreated meniscal, tetra‐PEG hydrogel and hydrogel–meniscal construct. (E) Live/dead assay showing the distribution and survival of rabbit chondrocytes within the tetra‐PEG hydrogel.

To further understand how tetra‐PEG bioadhesive and meniscal tissue were merged, we looked at the hydrogel‐tissue contact using scanning electron microscopy (SEM). The structural predictions of the hydrogel‒meniscal interfaces were confirmed by SEM images, which showed a tight and smooth contact between the surrounding meniscal tissues and hydrogels (Figure 4A). Furthermore, the observation clearly showed the penetration of the tetra‐PEG bioadhesive into the surface of the meniscus tissue, forming a tightly bound layer. The meniscus fibers were physically intertwined with the polymeric chains, confirming the adhesive mechanism of the tetra‐PEG hydrogel. This mechanism involves synergistic effects of chemical anchoring and physical chain entanglement to firmly bind the meniscus.

EDX component analysis by scanning electron microscope (EDX) elemental mappings and EDX spectrum revealed the existence of C, O, N, Ca, P, and S (Figure 4B,C), further indicating the strong adhesion onto the meniscus wherever gel flows through the meniscus. In addition, FTIR spectra provided the additional evidence for these strong hydrogel‒meniscus bonding interactions. Figure 4D shows that the tetra‐PEG hydrogel had a characteristic peak that was attributable to residual C=O of NHS‐ester group at 1625 cm^−1^. After treatment of the meniscus with the tetra‐PEG bioadhesive, this disappeared peak indicated the sufficient reaction of residual NHS‐ester group with the meniscus tissues. This discovery not only validates the adhesive mechanism, attributed to the synergistic effects of robust chemical anchoring between the amine‐rich proteins and NHS‐active ester hydrogels, and strong physical entanglements between the meniscus fibers and polymeric chains, but also indicates that the enhanced hydrogel‐tissue adhesion of the bioadhesive system can increase interfacial toughness by releasing a significant amount of mechanical energy when subjected to external stressors after application.

In addition, we found that when the primary rabbit chondrocytes were cultured in this tetra‐PEG bioadhesive for consecutive 7 days, the percentage of live chondrocytes was found to be more than 99% (Figure 4E), exhibiting the excellent cell activity, growth and proliferation of within the tetra‐PEG bioadhesive (excessive cell survival causes stacking, making accurate quantification challenging). The high cell survival and smooth migration observed suggest that chondrocytes can seamlessly transition between the tetra‐PEG bioadhesives and meniscus tissues. Consequently, as the tetra‐PEG bioadhesive degrades gradually, the extracellular matrix can fill meniscus cracks, aligning with the tissue repair process. Thus, this tetra‐PEG bioadhesive not only demonstrates robust adhesive strength for effective bonding and sealing but also offers supportive assistance in promoting cell migration and meniscus regeneration.

### 2.5 Molecular mechanism that promotes meniscus repair of tetra‐PEG bioadhesive for multi‐omics analyses

**FIGURE 5:**
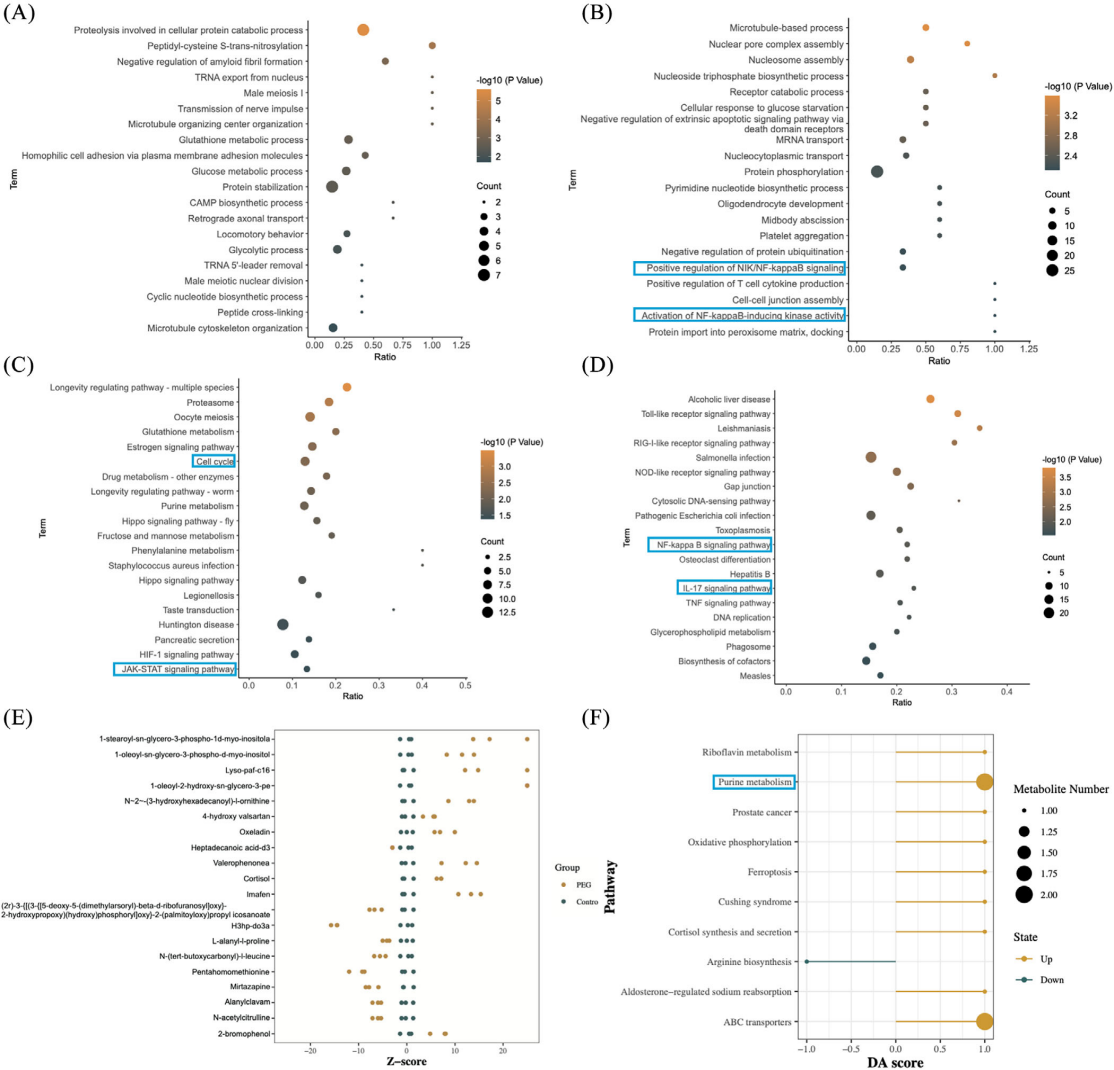
Bioinformatics analysis of the tetra‐poly (ethylene glycol) (PEG) promoting meniscus repair results on the basis of proteomics and metabolomics data. (A) Upregulated down‐regulated differential proteins (DEPs) Gene Ontology (GO) enrichment. (B). Down‐regulated DEPs using GO enrichment. (C) Up‐regulated DEPs KEGG enrichment. (D) Down‐regulated DEPs KEGG enrichment. (E) *Z*‐score plot of the down‐regulated differential metabolites (DEMs). Yellow and green colors indicate positive and negative correlations, respectively. (F) Map of the DEMs metabolic pathway enrichment analysis abundance score.

To further explore the molecular mechanism of tetra‐PEG bioadhesive for promoting the meniscus repair, the rabbit meniscus cells were processed in the tetra‐PEG and the samples were collected after 24 h for multi‐omics analysis. Volcano plots showed 236 up‐regulated and 437 down‐regulated differential proteins (DEPs) after treatment (Figure S14). differential protein KEGG enrichment analysis (KEGG) pathway enrichment analysis DEPs included cellular processes, environmental information processing, genetic information processing, human diseases, metabolism, and organismal systems (Figure S15). KEGG pathway enrichment analysis DEPs expressed in each group were represented as the heatmap (Figure S16). GO analysis of up‐regulated DEPs showed the up‐regulation of cellular transcriptional functions such as TRNA export from nucleus, CAMP biosynthetic process, and cyclic nucleotide biosynthetic process (Figure 5A). GO analysis of down‐regulated DEPs revealed the functional changes in post‐transcriptional modification of proteins and inhibition of inflammation‐related functions, such as protein phosphorylation, positive regulation of NIK/NF‐κB signaling pathway (NIK)/NF‐κB signaling pathway (NF‐κB) signaling, activation of NF‐κB‐inducing kinase activity and positive regulation of T‐cell cytokine production (Figure 5B). KEGG analysis of up‐regulated DEPs showed the up‐regulation of cellular transcriptional signaling pathways, such as cell cycle, hippo signaling pathway, HIF‐1 signaling pathway, and JAK‒STAT signaling pathway (JAK‒STAT) signaling pathway (Figure 5C). KEGG analysis of down‐regulated DEPs revealed the signaling pathway changes in inhibition of some important protein receptors and inhibition of inflammation‐related functions such as Toll‐like receptor signaling pathway, RIG‐I‐like receptor signaling pathway, NOD‐like receptor signaling pathway, NF‐κB signaling pathway, interleukin‐17 (IL‐17) signaling pathway, and tumor necrosis factor signaling pathway (Figure 5D). The aforementioned results indicate that the tetra‐PEG bioadhesive may enhance meniscus repair post‐injury, potentially by facilitating the transcription and translation processes of chondrocytes while suppressing the transcription of inflammatory‐related pathways within the intracellular environment.

After treating rabbit meniscus cells with tetra‐PEG bioadhesive for 24 h to uncover more useful application mechanisms, we carried out non‐targeted metabolomic analysis to further confirm the accuracy of the experimental data. All metabolites in the control and treatment groups were detected and classified into phytochemical compounds, others, lipids, and compounds with biological roles (Figure S17). Volcano plots show 57 up‐regulated and 31 down‐regulated differential metabolites (DEMs) after treatment (Figure S18). Clustering analysis DEMs expressed in each group are represented as the heatmap (Figure S19). The relative content of metabolites at the same level is measured using the *Z*‐score (standard score), a number translated based on the relative concentration of metabolites. When the absolute value of the *Z*‐score is greater than 20, it is displayed at the boundary position of the *x*‐axis. The *Z*‐score graph of the DEMs of each comparison group in this project such as Lyso‐paf‐c16 and Oxeladin is as follows (Figure 5E). Using a pathway‐based approach, differential abundance score can be used to assess metabolic alterations. The overall variations of all the metabolites in a pathway are captured by the differential abundance score. The top 10 metabolic pathways, including purine metabolism, aldosterone‐regulated salt reabsorption, and ABC transporters, that were significantly enriched in each comparison group underwent differential abundance score analysis (Figure 5F). The purine metabolism pathway, where inosine and sulfuric acid are located, was found to have an important role by functional enrichment analysis.

### 2.6 Rapid and suture‐independent bonding of meniscus tissues

**FIGURE 6:**
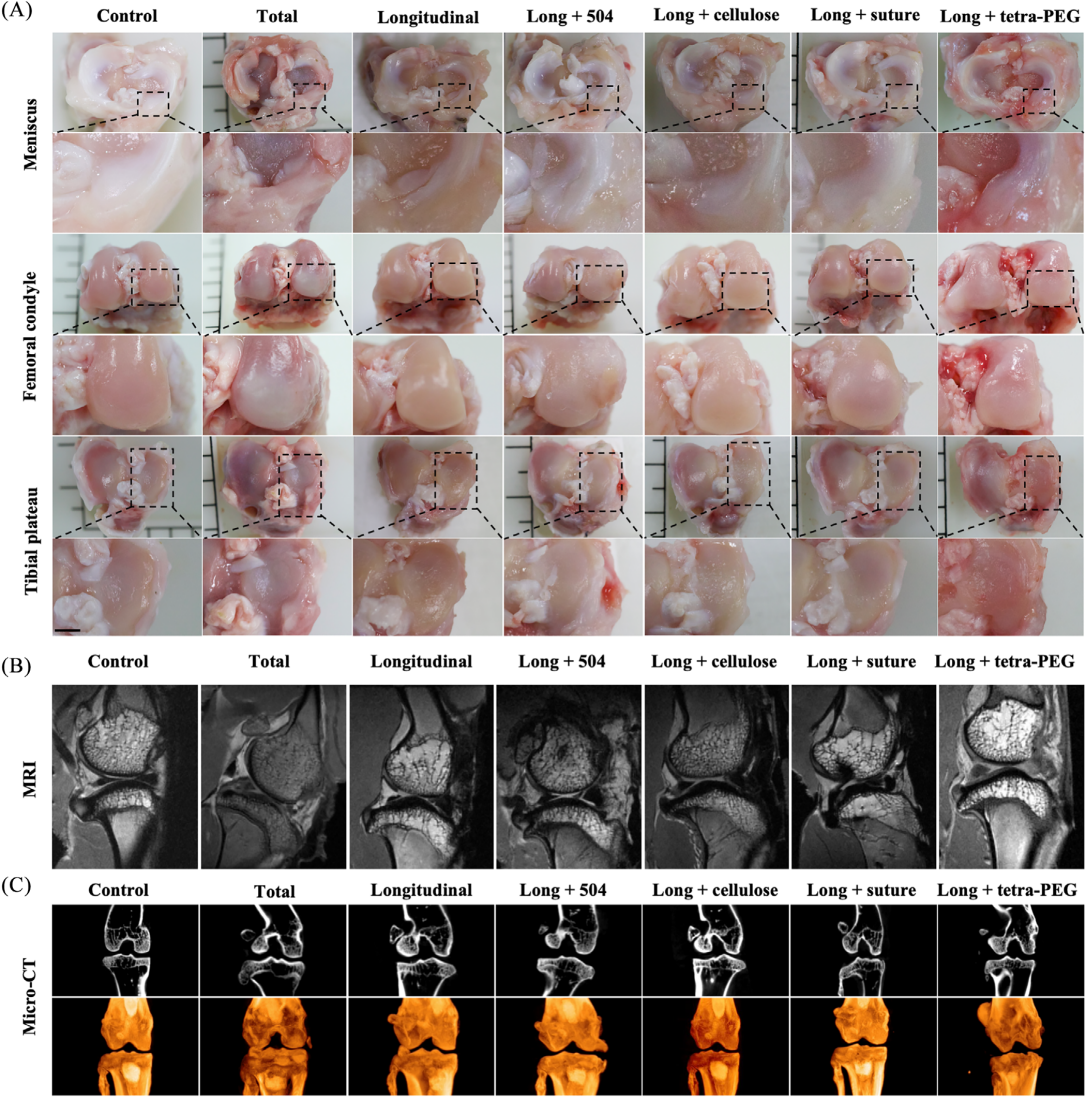
General evaluation of treatment based on various adhesive in a rabbit model. (A) The repair level of meniscal injury, and protective effect of medial femoral condyle and tibial plateau cartilage. (B) Magnetic resonance imaging (MRI) showing the level of cartilage protection. (C) Observation of cartilage protection level and bone flab formation using micro‐computed tomography (CT) (scale bar = 0.5 cm).

On the basis of the in vitro results of tetra‐PEG bioadhesive, verification was done using a severe meniscus injury model created by a longitudinal incision (a most common type of clinical meniscal injuries). Seven groups were used to determine the therapeutic impact, including: sham group, total group (complete removal of the rabbit's meniscus), longitudinal group, long + 504 group (504 applied to the meniscus longitudinal injury models), long + cellulose group (cellulose applied to the meniscus longitudinal injury models), long + suture group (surgery suture applied to the meniscus longitudinal injury models), and long + tetra‐PEG group (tetra‐PEG bioadhesive applied to the meniscus longitudinal injury models). The reconstructed knee joints were removed from the rabbits after 3 months so they could be examined grossly, histologically, and for biochemical and biomechanical testing. The total group and the longitudinal group showed gross observation of the knee joints with significant meniscus injury features, such as erosions and huge defects, with increasing cartilage loss over time. The long + tetra‐PEG group had the most pronounced cartilage injury (Figure 6A). The aforementioned findings, which revealed substantial cartilage damage in the total group and longitudinal group, were corroborated by magnetic resonance imaging (MRI) analysis. Long + suture group cartilage displayed a significantly smoother surface, and the signal intensity of the cartilage tissue was comparable to the control group (Figure 6B). Next, we examined subchondral bone remodeling and osteophyte development to corroborate the therapeutic efficacy of tetra‐PEG bioadhesive. The impact of the therapy on osteophyte development and tibia subchondral bone remodeling was evaluated using micro‐computed tomography (CT). The long + tetra‐PEG group exhibited less bone fragmentation and more intact subchondral bone biology, as can be shown in Figure 6C.^23^

### 2.7 Protect cartilage function and inhibit degeneration

**FIGURE 7:**
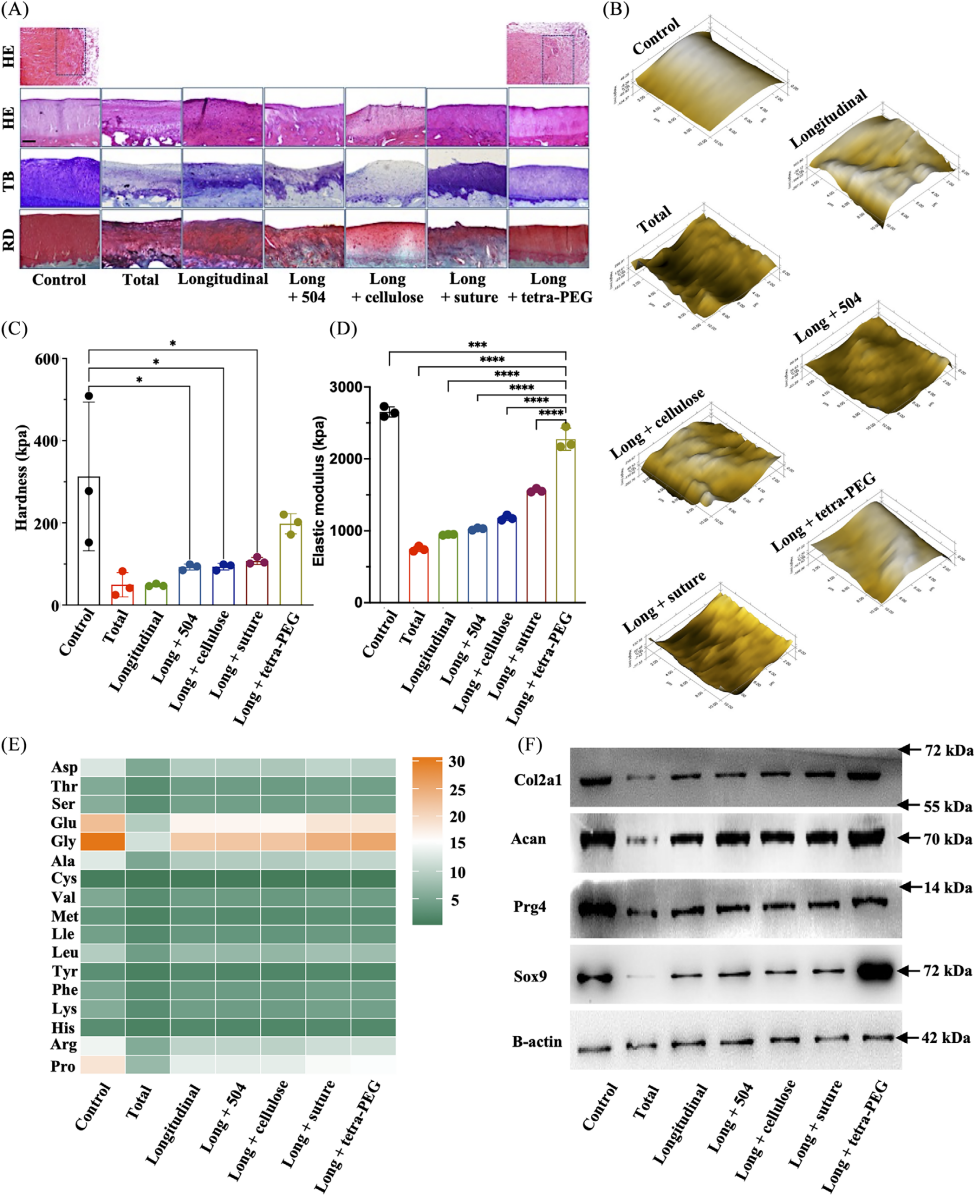
General evaluation of treatment using tetra‐poly (ethylene glycol) (PEG) bioadhesive in a rabbit model. (A) Levels of meniscus and cartilage protection using pathological staining methods. (B‒D) Evaluation of stiffness, elastic modulus and hardness of the cartilage using nanoindentation. (E) Analysis of the amino acid composition and expression levels of cartilage using amino acids. (F) Levels of cartilage‐expressed collagen‐associated protein by Western blotting (scale bar = 100 μm).

A histological investigation utilizing hematoxylin and eosin (H&E), toluidine blue (TB), and Saffron solid green staining provided additional confirmation for these offensive discoveries. The meniscus and cartilage areas in the long + tetra‐PEG group had consistent findings with reasonably homogenous, hyaline, and cartilage‐like histological characteristics, demonstrating a robust cartilage‐specific extracellar matrix (ECM) staining and equivalent cartilage thickness. According to Figures 7A and S20, the findings of H&E staining revealed the structure of the healed region following meniscus damage in the tetra. By contrast, the total group and the longitudinal group showed considerable variability among individuals compared to the control group. The cartilage and meniscus areas both had visibly damaged tissue, thin cartilage depth, and rough surfaces. Therefore, due to the insufficient capacity for self‐repair, meniscal tears are incapable of preserving the integrity of articular cartilage. Overall, the long + tetra‐PEG group had considerably higher cartilage histological appearance than other groups.

The biomechanical characteristics of the articular cartilage were revealed by the nanoindentation. Higher hardness and elastic modulus in the long + tetra‐PEG group in comparison to the longitudinal group suggested better biomechanical properties of regenerated cartilage similar to control cartilage (Figure 7B‒D). Thus, tetra‐PEG bioadhesive outperformed typical surgical suture therapy in terms of chondroprotection for meniscus repair, according to macro‐ and microscopic observations and biomechanics analysis. Using an amino acid analyzer, articular cartilage amino acids were analyzed qualitatively and quantitatively. The results showed that the amino acid composition and expression level of long + tetra‐PEG group were closest to those of control group, especially for the glycine (representing the collagen content) (Figure 7E). Consistent with the Western blot results, long + tetra‐PEG group could increase the protein expression of SOX9, Prg4, aggrecan, and collagen II that were similar to the control cartilage (Figure 7F).

### 2.8 Inflammation regulation protects cartilage function

**FIGURE 8:**
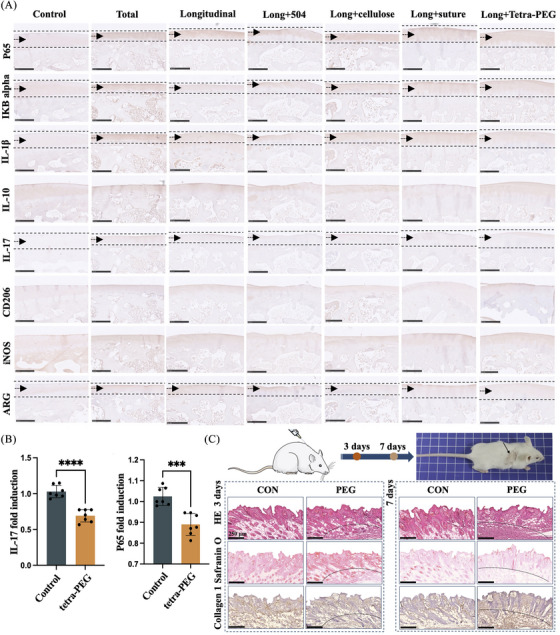
Inflammatory regulatory mechanism using tetra‐poly (ethylene glycol) (PEG) bioadhesive in a rabbit model. (A) Immunohistochemical staining to verify inflammatory regulatory mechanism. (B) RT‐PCR to verify inflammatory regulatory mechanism. (C) Schematic procedure for subcutaneous implantation of tetra‐PEG hydrogel and representative histological images of hematoxylin and eosin (H&E), Safranin O, and type I collagen staining of the skin in vivo. The dotted line marks the subcutaneous implantation region of hydrogel (scale bar = 250 μm).

Upon further investigation into the animal model of inflammation, it was observed that the tetra‐PEG group exhibited significant suppression of the IL‐17‐mediated inflammatory pathway and modulation of the NF‐κB inflammatory signaling pathway. Additionally, varying degrees of regulation were observed in the M1/M2 cells conversion, consistent with our sequencing results (Figure 8A). Furthermore, consistent results were obtained from the mRNA expression levels of meniscus chondrocytes influenced by the tetra‐PEG bioadhesive (Figure 8B). An experimental model of static subcutaneous implantation was employed to assess the biosafety of tetra‐PEG bioadhesive. Shortly, tetra‐PEG hydrogels were implanted subcutaneously into nude mice and harvested after 3 days/7 days to assess the extent to which the hydrogels stimulated acute inflammation and the ability to provoke normal tissue. Pathological staining results showed that the hydrogels activated the recruitment of neutrophilic granulocyte on day 3 and gradually increased on day 7, and normal skin morphology and extracellular matrix were not significantly agitated (Figure 8C). These results revealed the excellent cytocompatibility and biosafety of tetra‐PEG hydrogel with a great prospect in soft tissue adhesion in vivo without triggering a persistent inflammatory reaction.

## 3 DISCUSSION

Despite much academic and industrial research into meniscus repair technologies and apparatus, partial meniscus repair, and chondroprotection continue to be clinical difficulties.^24^, ^25^ Meniscal damage can result in arthritis and cartilage degradation.^26^, ^27^, ^28^ Fifty million Americans alone suffer from various forms of arthritis, thus finding techniques to prevent or delay the onset of arthritis from meniscal abnormalities is essential.^1^ Current surgical techniques only provide temporary symptom alleviation and have a limited capacity for regenerating tissue.^29^ Techniques for tissue engineering are becoming available as alternatives for meniscus and cartilage repair. There are currently several cell‐based and tissue‐engineered products in meniscal tear clinical studies, providing new opportunities for meniscus regeneration.^30^, ^31^, ^32^, ^33^

Furthermore, various stringent requirements such as biological variability, biomimicry, implant integration and protection, inflammation and immunogenicity, regulatory concerns, complexity of administration, as well as the multifaceted mechanism further limit its efficacy in meniscus regeneration. Therefore, exploitation of an innovate therapeutic strategy with simple, safe, and high‐efficient surgery therapy is urgently needed for clinical complex meniscus injury. Alternatively, tissue adhesives have been developed to repair tissue damage without the need for surgical sutures. However, existing tissue adhesives often require harsh UV irradiation, prolonged steady pressure application (>3 min), and/or a strict dry environment, significantly limiting their effectiveness and utility in true clinical applications.

In this research, we present a tetra‐PEG bioadhesive that combines simple application, high safety, rapid curing time, easy injectability, excellent mechanical strength, and robust tissue adhesion. This facilitates successful bonding of meniscus tissues during surgery without the need for sutures. In vivo rabbit models demonstrate the effectiveness of the tetra‐PEG bioadhesive as a promising solution for rapid and strong bonding of meniscus tissues, surpassing commonly used FDA‐approved adhesives such as fibrin glue and cyanoacrylate glue, even in wet environments.

While we have not conducted in vivo testing specifically for radial tear meniscus repair, it is widely acknowledged that radial tears are challenging to repair with sutures, leading to frequent failures, particularly with the medial meniscus, despite the development of various techniques in recent years.^34^, ^35^, ^36^ Similarly, complex meniscus injury also cannot be repaired in clinical operations, and patients often undergo partial or total excision.^37^, ^38^ Despite sharing compositional similarities with commercial polyethylene glycols (COSEAL), the bioadhesive exhibits distinct advantages, including ultrafast gelation time, extended degradation period, and robust adhesion strength within the knee joint cavity. Furthermore, its ability to deliver nutrients in a moisturizing environment could facilitate the treatment of complex and hard‐to‐reach meniscal injuries, representing a significant technological advancement.^39^, ^40^, ^41^, ^42^ While the efficient bonding of multiple organs such as skin, heart, liver, spleen, and vessel injuries has been demonstrated, the preliminary adhesive practicability and availability of tetra‐PEG bioadhesive have also been validated through in vivo meniscus injury repair in weight‐bearing regions of swine models (Figure S21).

Considering its explicit structure, mechanical tissue integrity, and easy usage, this biocompatible adhesive could potentially encapsulate cells, biological factors, and drugs to enhance cartilage repair and functional reconstruction. This highlights the advanced advantages of our highly versatile multifunctional biomaterial, presenting an intriguing concept for achieving the ultimate goal of tissue engineering applications. Hence, armed with advanced understanding of the properties and functionalities of tetra‐PEG hydrogel, we are confident that this bioadhesive holds substantial therapeutic promise for treating various cartilage tissues as it transitions from laboratory research to clinical application.

## 4 METHODS

### 4.1 Materials

Tetra‐PEG‐OH (*M*_w_ = 10 kDa, *M*_w_/*M*_n_ = 1.03) and tetra‐PEG‐NH_2_ (*M*_w_ = 10 kDa, *M*_w_/*M*_n_ = 1.03) were purchased from Xiamen SINOPEG Biotech Co., Ltd. 1‐(3‐(Dimethylamino) propyl)−3‐ethylcarbodiimide hydrochloride, dimethylamino‐pyridine (DMAP), and N,Nʹ‐disuccinimidyl carbonate were purchased from Energy Chemical.

### 4.2 Synthesis of tetra‐PEG‐SC polymer

Tetra‐PEG‐OH (0.1 mmol, 1 g), N,Nʹ‐disuccinimidyl carbonate (4 mmol, 1.03 g), and DMAP (4 mmol, 0.45 g) were dissolved in 50 mL of dry CH_2_Cl_2_. The system was stirred for 12 h and then directly washed with 2 M HCl aqueous solution, saturated NaCl aqueous solution and deionized water for several times, and dried over anhydrous Na_2_SO_4_. The resulting product underwent three more precipitation steps into diethyl ether to produce tetra‐PEG‐SC white powder.

### 4.3 Preparation of tetra‐PEG hydrogel

In two sample bottles, 5, 10, 15, and 20 wt% of tetra‐PEG‐NH_2_ and tetra‐PEG‐SC polymers, respectively, were dissolved in PBS (pH 7.4). Then, a dual syringe was used to combine two components to create the injectable tetra‐PEG hydrogel. The gelation time was the period of time during which the solutions were not flowing backward. We have developed a strong hydrogel bioadhesive as a sutureless sealant (tetra‐PEG‐SC). Once they were synchronously sprayed onto the injured meniscus, tetra‐PEG‐NH_2_ polymer's amine groups swiftly connected with tetra‐PEG‐SC polymer's NHS‐ester to create dependable crosslinking networks and crosslinking amide connections, which supported the material's exceptional mechanical toughness. Tetra‐PEG‐SC aqueous solutions entered the irregularly shaped meniscus tissue cavities and simultaneously achieved tissue adhesion. They then quickly reacted with the amine groups present in the tissue proteins or the nearby cartilage surfaces, firmly attaching to the meniscus to withstand hydraulic and internal pressure on the knee without the need for additional sutures.

### 4.4 Scanning electron microscope observation

A scanning electron microscope was used to look at the produced cartilage samples' surfaces (HITACHI). The gold‐palladium coating of the dehydrated samples was subsequently applied using a Hitachi S‐3400N ion sputter.^43^

### 4.5 Water retention capacity

The weight method was used to calculate the hydrogels' ability to retain water across a range of time periods at 25°C and 50% humidity. The hydrogel samples with different solid contents were placed on a dry glass platform, and the samples were taken every 15 min and photographed.

### 4.6 Rheology

Thermo Hanke plate geometry (35 mm diameter) and 25°C were used for the rheometer. With a spacing of 1.5 mm, the hydrogels were made and placed on the plate. We conducted an amplitude sweep before to the testing to identify the region of linear viscoelasticity where the storage modulus was independent of strain amplitude. Using a frequency range of 100‒0.1 rad/s, the sample was measured.

### 4.7 Bursting pressure experiments

To remove any excess fat, a 4 cm by 4 cm square of hog casing was cleaned and cut. The measurement tool, which contained water and was attached to a syringe pump, was coupled to the hog casing. A 1‐mm incision was made in the surface of the hog casing, and then hydrogels started to develop there. The hydrogels' full gelation allowed for the measurement of the burst pressure.

### 4.8 Adhesive strength

The porcine skin was used to gauge the adhesive strength. First, porcine strips with dimensions of 30 mm by 10 mm were produced. Inject the glue onto the surface of the pig skin and let it rest for 10 min. The pork skin is curled and folded at various angles.^44^, ^45^

### 4.9 Blocking PE/glass bottles

A 0.5 cm of diameter circular hole was drilled in the PE/glass bottle body contained double distilled water (DDW). After sealing the circular hole with hydrogel for 1 h, the sealing performance around the hole was observed.

### 4.10 Multi‐organ rupture bonding assay in vivo

The model animals were 3‐month‐old rabbits, which were anesthetized and fixed, and then rupture models were created on the skin, heart, liver, spleen, and blood vessels, which were glued with tetra‐PEG bioadhesive for 1 s and photographed.

### 4.11 Biocompatibility

Rats (*n* = 3) were implanted with the hydrogels in their subcutaneous tissue as‐prepared (*n* = 3). Rats were put to sleep before the hydrogels with surrounding tissue were retrieved after being implanted for 3 and 7 days. These samples were meticulously stripped of superfluous tissue after being thoroughly cleaned in distilled water.

### 4.12 Cell survival and distribution

The location and survival of rabbit chondrocytes in hydrogel were verified using the live/dead assay. Briefly, after incubation for 7 days, the cell culture inserts were removed. Confocal microscopy was used to observe the distribution and morphology of live (green if the cell viability was greater than 70%) and dead (red fluorescence) cells (excitation: 488 or 568 nm).^46^

### 4.13 Meniscus simulated adhesion experiment in vitro

The meniscus of a 3‐month‐old rabbit was taken, completely transected, re‐glued using sutures or various biological glues, and placed in a Petri dish containing PBS for 1 h to simulate a confined and moist environment in the joint cavity, and then the maximum weight‐bearing force for re‐rupture of the meniscus was detected using the weighing method and clamping the ends of the meniscus using hemostatic forceps.

### 4.14 FTIR

The meniscus samples were freeze‐dried and ground into powder using an automatic grinder. By drop‐casting sample films from a polymer solution in chloroform onto a KBr plate and then drying them with an infrared laser, infrared spectra were captured on an Excalibur Series FTIR spectrometer.^47^

### 4.15 Under water using adhesive in vitro

The bonded meniscus was fixed in a petri dish, and the meniscus was rinsed continuously under the operating water pressure using an arthroscope to observe the firmness of the bioadhesive. A hydraulic pressure gauge (*n* = 3) measured the hydraulic pressure.

### 4.16 Multi‐omics analysis

Rabbit meniscus cells were processed in the tetra‐PEG and collected after 24 h (*n* = 3), and then sent to the laboratory of China Huada Company for proteomics and metabolomics experimental procedures, followed by protein and metabolite processing. Data comparison and database construction are all completed by the company's laboratory. We used R (https://cloud.r‐project.org), Dr. Tom (https://biosys.bgi.com), STRING (https://cn.string‐db.org), and Cytoscape (https://github.com/cytoscape) for subsequent bioinformatics analysis.

### 4.17 Animal study

Longitudinal with radial incision was used to cause a significant rabbit meniscus damage in adult female New Zealand rabbits weighing 3.0‒3.5 kg. Then, the long + tetra‐PEG group specified meniscus damage models were exposed to the tetra‐PEG bioadhesive. We used six groups, including control group, total group (complete excision of the rabbit meniscus), longitudinal group, to determine the mechanism of the therapeutic effect (no operation on the meniscus longitudinal injury models), long + 504 group (504 applied to the meniscus longitudinal injury models), long + cellulose group (cellulose applied to the meniscus longitudinal injury models), and long + suture group (surgery suture applied to the meniscus longitudinal injury models). After treatment for 3 months, all the rabbits were sacrificed and the joints were collected for further study.

### 4.18 Cartilage MRI

The MRI of cartilage was performed on the Bruker biospec 7 T machine (Bruker). All the rabbits underwent 7.0‐T MRI scanning of the knees for analysis of cartilage repair at 3 months after treatment. The T2 turbo and T2 mapping sequences were acquired using the methods as previously reported.^48^

### 4.19 Micro‐CT of subchondral bone CT

All rabbits' knee joints were removed after 3 months of treatment and preserved in 4% formaldehyde for 48 h. A micro‐CT scanner was used to scan the region 3 mm from the plateau at the proximal end of the tibia (Siemens).^49^

### 4.20 Macrography and histological analysis

According to the manufacturer's instructions, type II collagen antibody immunostaining, Safranin O‐fast green (SO&FG), TB, and H&E staining were all done for histological analysis.

### 4.21 Nanoindentation analysis

Based on previously published techniques, nanoindentation was utilized to assess the biomechanical characteristics of cartilage tissues in each group.^50^ The trapezoidal loading function was applied to each indentation site, which were the loading phase for 10 s, the maintenance phase for 2 s, and the unloading phase for 10 s, and the cartilage surface displacement was 500 nm.^51^

### 4.22 Amino acid composition analysis

An amount of 1 mL of methanol was used to dissolve 1 mg of the material. The order of retention duration and spectrum similarity were necessary for the identification of each amino acid. The automated amino acid analyzer was loaded with resuspended amino acids after drying in 200 L of 0.01 N HCl (Hitachi High‐Technologies, model L‐8900).^52^

### 4.23 Statistical analysis

The mean and standard deviation are used to show the data (standard deviation). The analysis of variance and unpaired Student's *t*‐test were used, respectively, to statistically compare the differences between two groups and between several groups. The entire data set was examined with SPSS software (v.18.0).

## AUTHOR CONTRIBUTIONS

J.Y., Y.C., and R.D. conceived the project and designed the study. J.Z. and H.W. performed experiments. S.S. and X.W. analyzed the data. B.X., X.W., and J.‐K.Y. provided experimental materials and scientific suggestions. J.Y., Y.C., and R.D. wrote the manuscript. All the authors reviewed the manuscript and discussed the work and have read and approved the final manuscript.

## CONFLICT OF INTEREST STATEMENT

The authors declare they have no conflicts of interest.

## ETHICS STATEMENT

All animal research received the nod of approval from Peking University's Third Medical School's Ethical Committee for Laboratory Animals (A2019030), and all operations were carried out in accordance with those standards.

## Supporting information



Supporting Information

Supporting Information

## Data Availability

All data are available from the corresponding authors upon request.
