# Supplementary material for: Robust tetra‐armed poly (ethylene glycol)‐based hydrogel as tissue bioadhesive for the efficient repair of meniscus tears
Source: MedComm (2020). 2024 Oct 24;5(11):e738. doi: 10.1002/mco2.738 (PMC11502715; doi:10.1002/mco2.738)
Supplement: Supplementary file 1 — Supporting Information [file MCO2-5-e738-s001.docx]

**Robust tetra-armed poly (ethylene glycol)-based hydrogel as tissue bioadhesive for the efficient repair of meniscus tears**

Jing Ye^1,2,†^, Yourong Chen ^1,2,†^, Ronghui Deng^1,2,†^, Jiying Zhang^1,2^, Hufei Wang^3,4^, Shitang Song^1,2^, Xinjie Wang^1,2^, Bingbing Xu ^1,2^*, Xing Wang^3,4^* and Jia-Kuo Yu^1,2,5,6,^*

^1^ Sports Medicine Department, Beijing Key Laboratory of Sports Injuries, Peking University Third Hospital. No.49, North Garden Road, Haidian District, Beijing 100191, China

^2^ Institute of Sports Medicine, Peking University. No.49, North Garden Road, Haidian District, Beijing 100191, China

^3^ Beijing National Laboratory for Molecular Sciences, Institute of Chemistry, Chinese Academy of Sciences, Beijing 100190, China

^4^ University of Chinese Academy of Sciences, Beijing 100049, China

^5^ Orthopaedic and Sports Medicine Centerl, Beijing Tsinghua Changgung Hospital, Tsinghua University, China

^6^ Institute of Orthopedic and Sports Medicine of Tsinghua Medicine, Tsinghua University, China

* Correspondence:

Jia-Kuo Yu

[yujiakuo@tsinghua.edu.cn](mailto:yujiakuo@tsinghua.edu.cn), yujiakuo@126.com

Xing Wang

[wangxing@iccas.ac.cn](mailto:wangxing@iccas.ac.cn)

Bingbing Xu

| xubingbing@hsc.pku.edu.cn |
| --- |

^†^ Jing Ye, Yourong Chen and Ronghui Deng contributed equally to this work.

Supplementary Figures

**FIGURE. S1** ^1^H NMR spectrum of tetra-PEG-SC polymer.

**FIGURE. S2** The 1H NMR and FT-IR spectra of tetra-PEG-NH2 and tetra-PEG-SC polymers.

**FIGURE. S3** Synthetic route of tetra-PEG bioadhesive

**FIGURE. S4** The strain amplitude sweep analysis at fixed angular frequencies of tetra-PEG hydrogels.

**FIGURE. S5** XRD analyses of tetra-PEG hydrogels.

**FIGURE. S6** FT-IR analyses of tetra-PEG hydrogels.

**FIGURE. S7** TGA analyses of tetra-PEG hydrogels.

**FIGURE. S8** DSC analyses of tetra-PEG hydrogels.

**FIGURE. S9** Enzymatic degradation of tetra-PEG hydrogels.

**FIGURE. S10** Live/dead assay showing the distribution and survival of rabbit chondrocytes within the tetra-PEG hydrogel.

**FIGURE. S11** CCK8 showing the distribution and survival of rabbit chondrocytes within the tetra-PEG hydrogel.

**FIGURE. S12 Burst pressure tests of tetra-PEG bioadhesive *in vitro*.** (**a**) Balloon and (b) Porcine small intestine burst pressure tests to determine the hydrogel-tissue binding strength of tetra-PEG bioadhesive with various concentrations of 5%, 10%, 15% and 20%.

**FIGURE. S13 Adhesion of tetra-PEG bioadhesive in vivo.** Injectable operation for the effective rupture bonding assay for skin, heart, liver, spleen, and blood vessels in vivo.

**FIGURE. S14** Representative volcano plots with a threshold of Fold change > 1.5 and a Q-value of 0.05 demonstrating changed protein expression in the designated clusters.

**FIGURE. S15** Analysis of the differentially expressed proteins using KEGG and KEGG enrichment (DEPs).

**FIGURE. S16** DEP clustering analysis.

**FIGURE. S17** Metabolites are categorized according to their compound class.

**FIGURE. S18** Volcano plots that serve as examples, displaying changed metabolites in the designated clusters.

**FIGURE. S19** Clustering analysis of differentially Metabolites (DEMs).

**FIGURE. S20** OARSI score.

**FIGURE. S21 Evaluation of meniscus injury treatment based on efficient, safe and sutureless tetra-PEG bioadhesive in a swine model. (a)** Schematic illustration of tetra-PEG bioadhesive in meniscus injury repair. **(b)** Photographs of *in vivo* operation of in meniscus defect repair using tetra-PEG bioadhesive. **(c)** One day after meniscus injury repair using tetra-PEG bioadhesive in a swine model.

**FIGURE. S22** Hg intrusion porosimetry to measure the porosity of biological glue.

**TABLE 1** Gel forming time quantitative table.


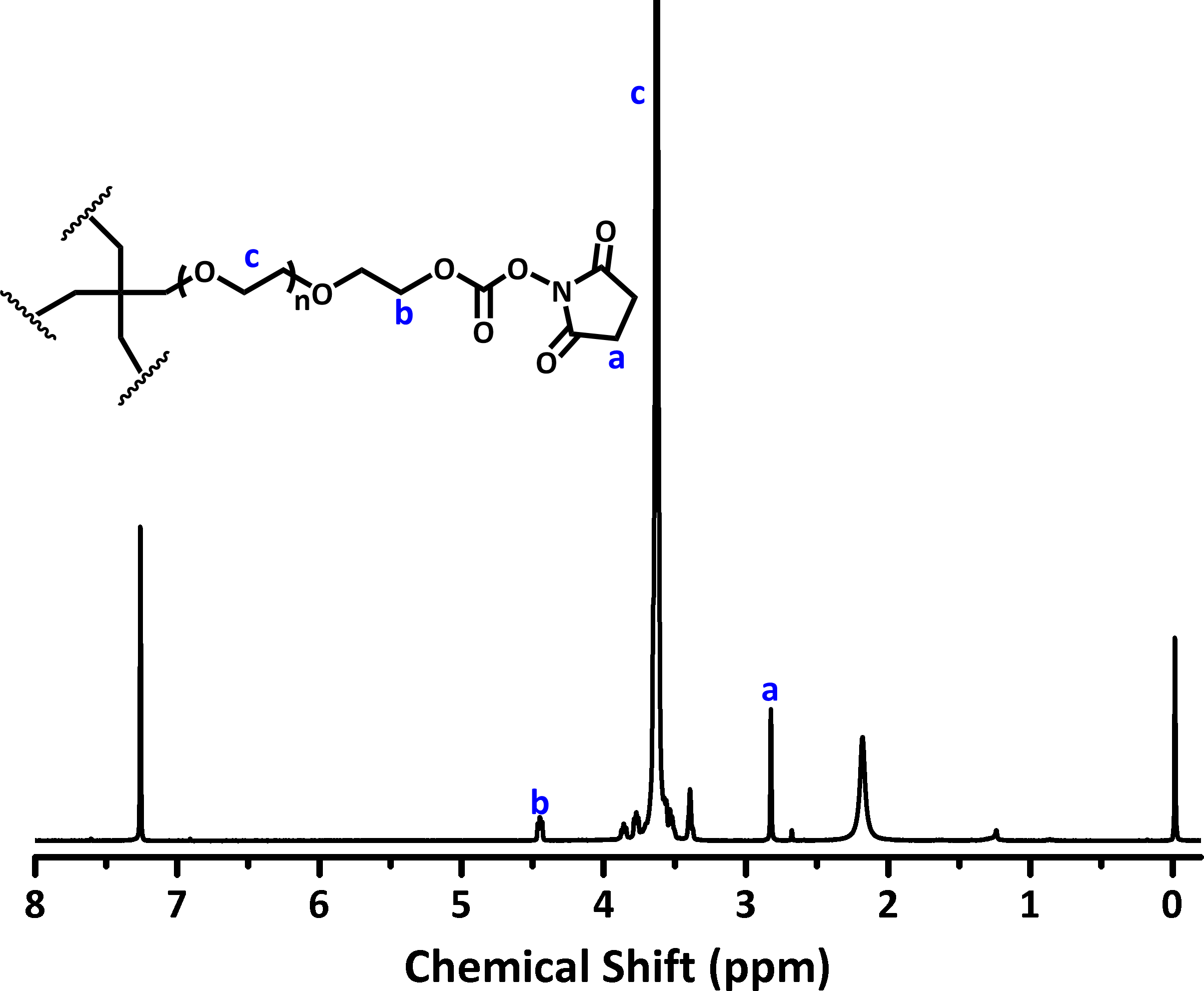


**FIGURE. S1** ^1^H NMR spectrum of tetra-PEG-SC polymer.


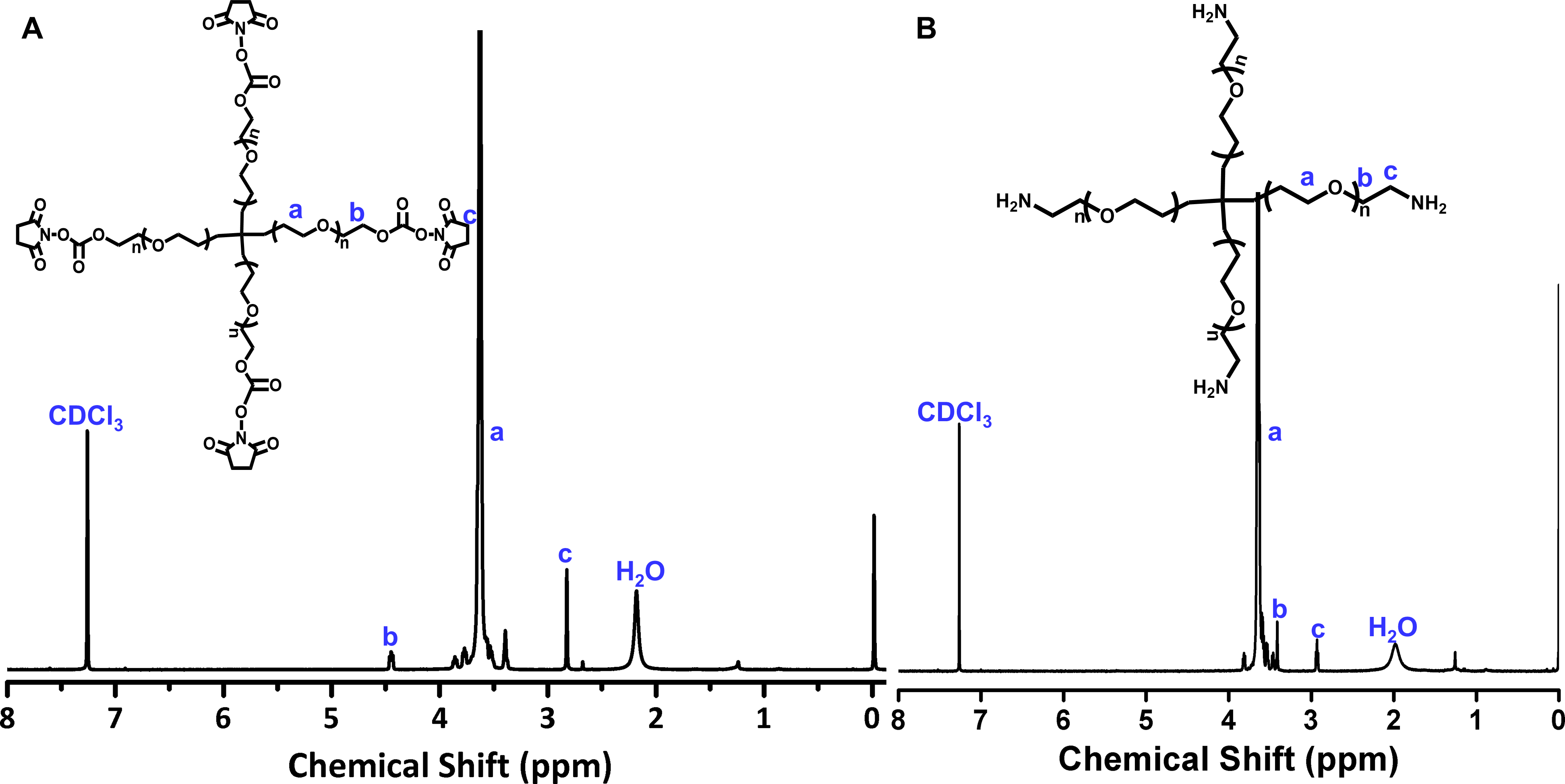


**FIGURE. S2** The 1H NMR and FT-IR spectra of tetra-PEG-NH2 and tetra-PEG-SC polymers.

**FIGURE. S3** Synthetic route of tetra-PEG bioadhesive


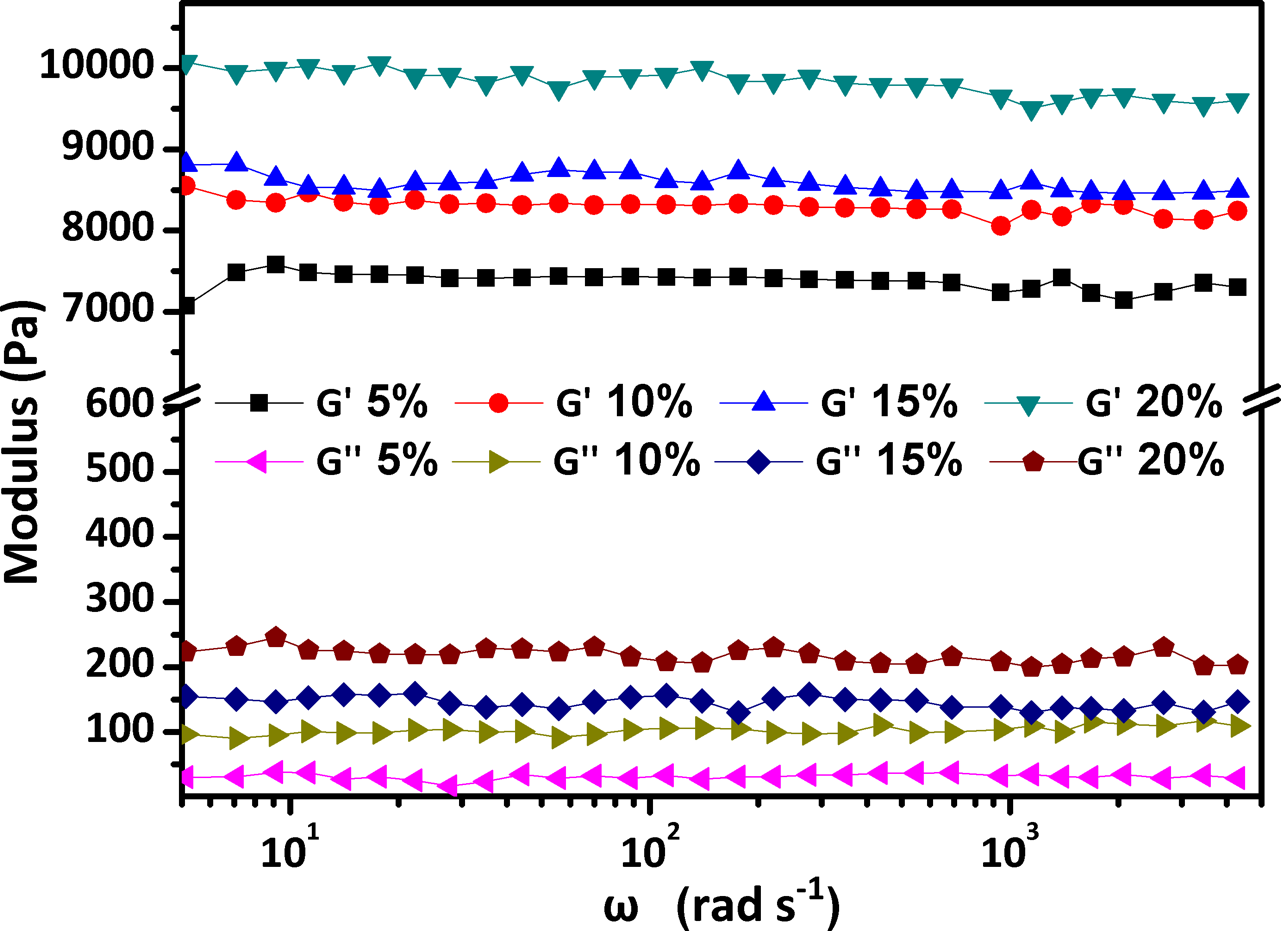


**FIGURE. S4** The strain amplitude sweep analysis at fixed angular frequencies of tetra-PEG hydrogels.


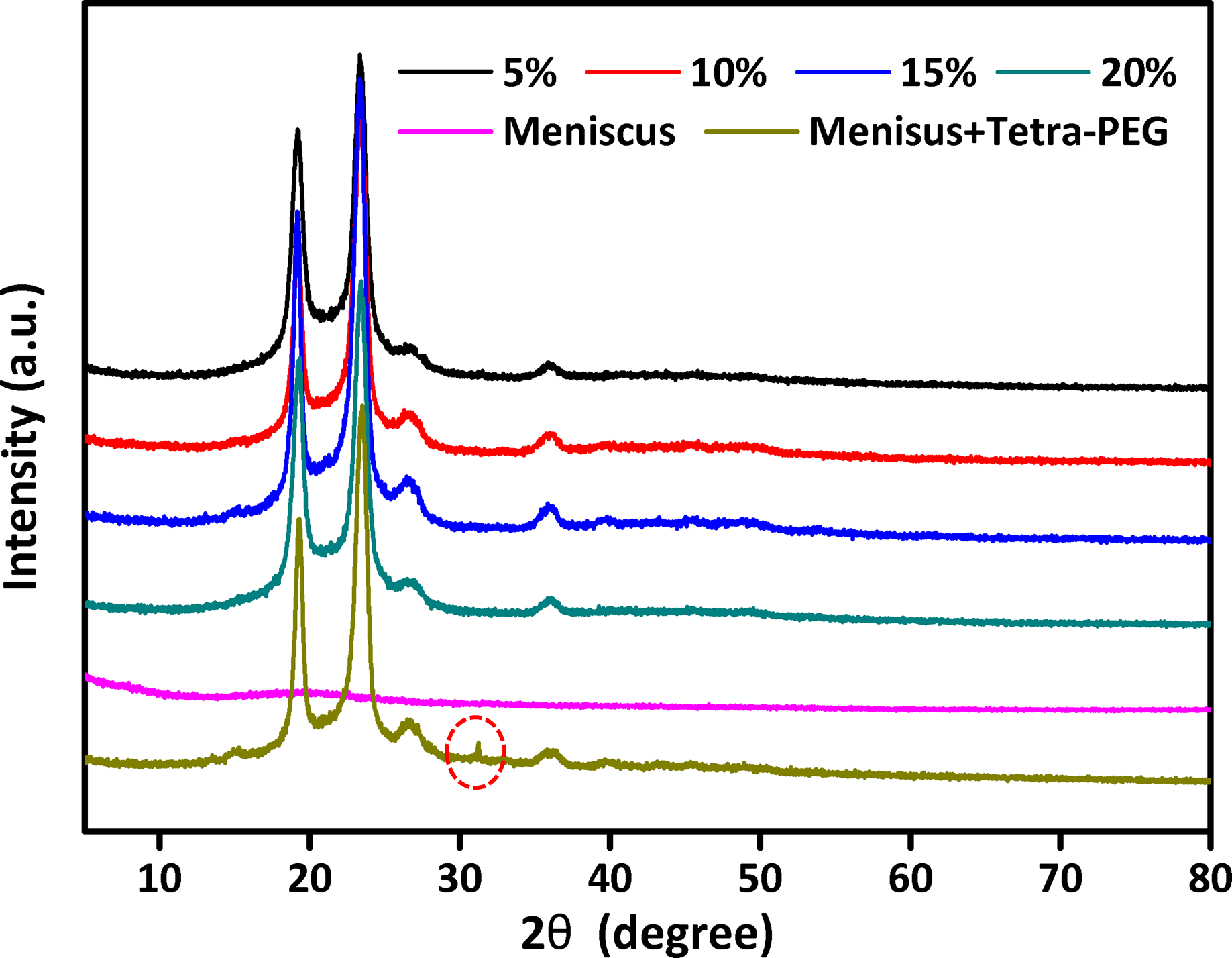


**FIGURE. S5** XRD analyses of tetra-PEG hydrogels.


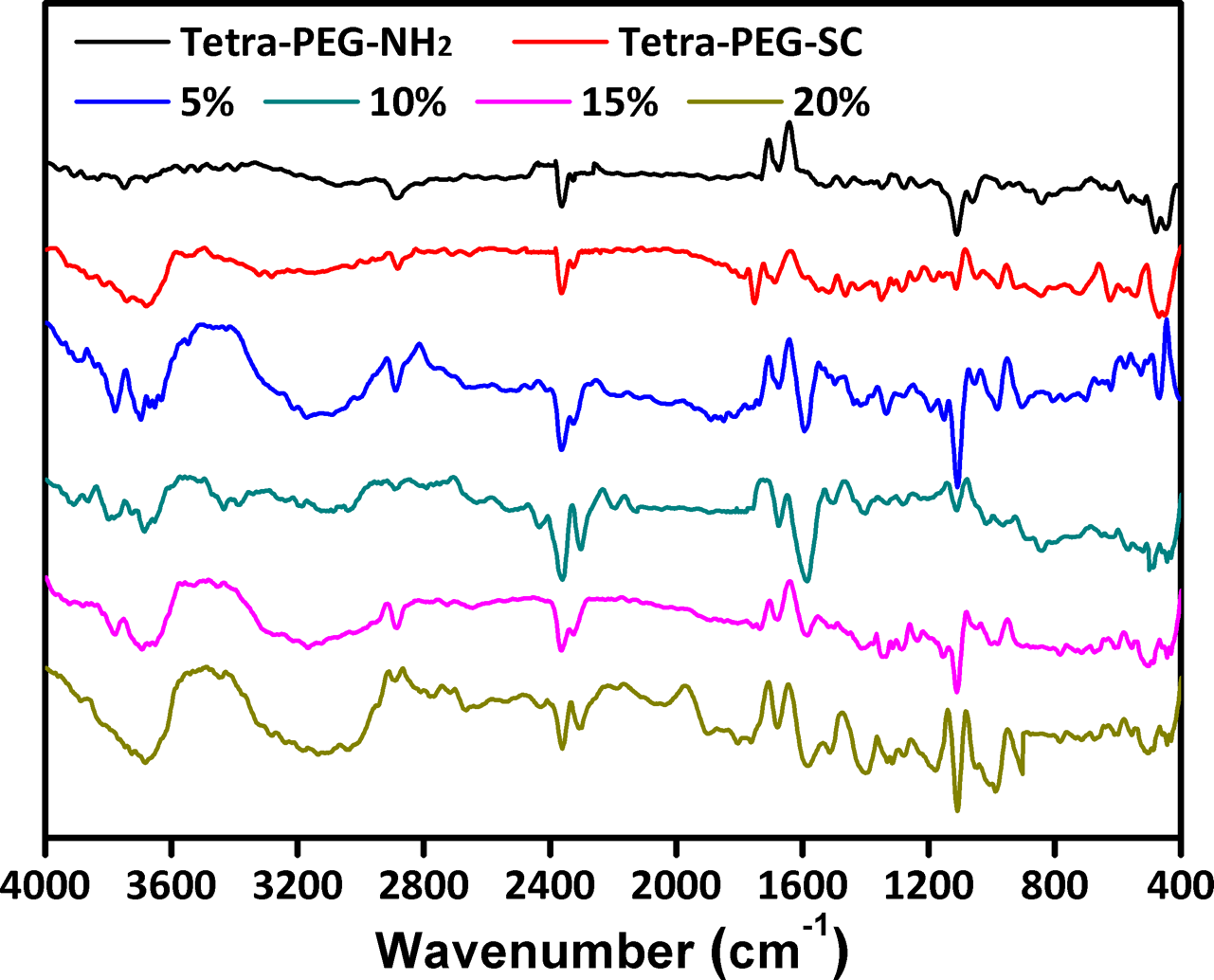


**FIGURE. S6** FT-IR analyses of tetra-PEG hydrogels.


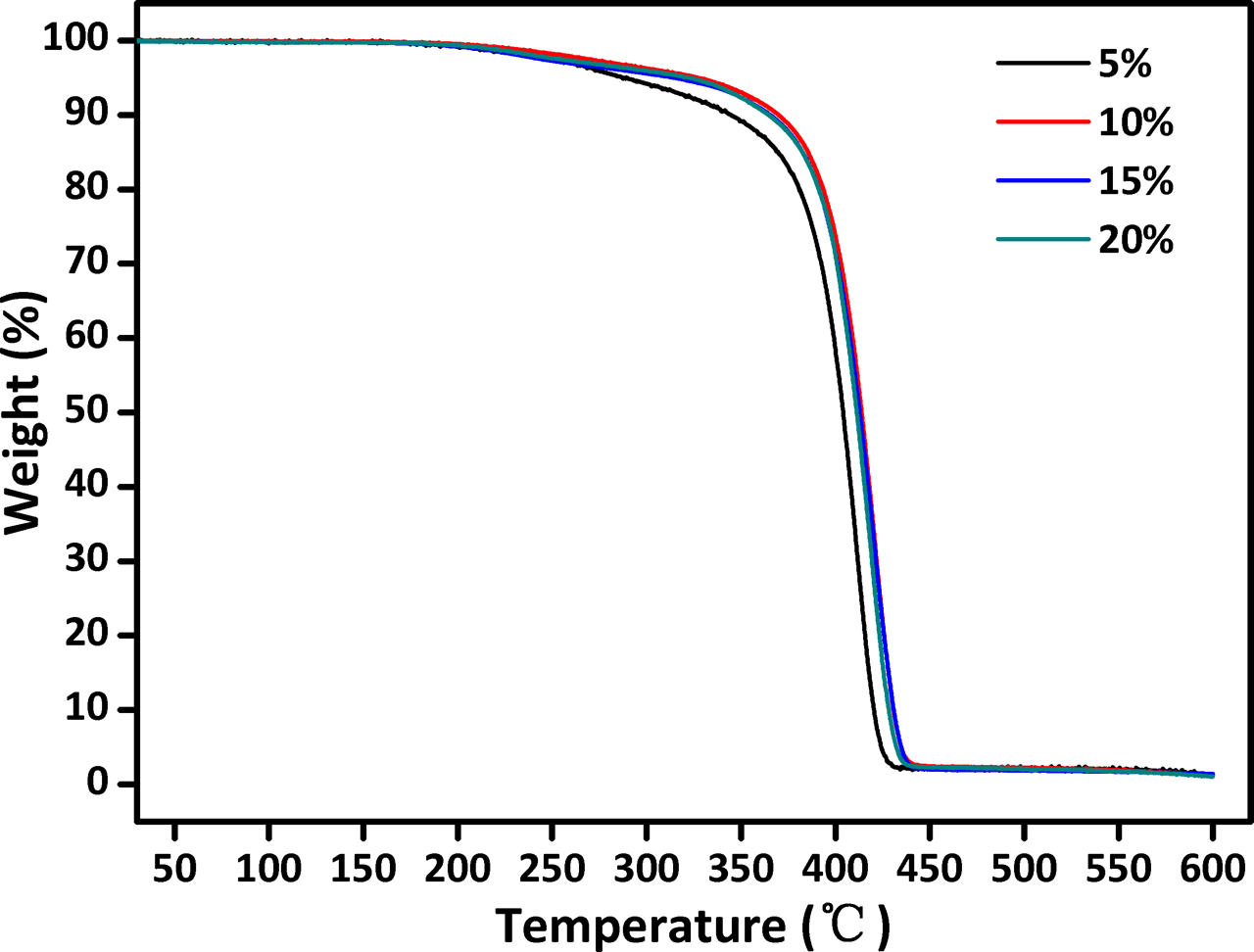


**FIGURE. S7** TGA analyses of tetra-PEG hydrogels.


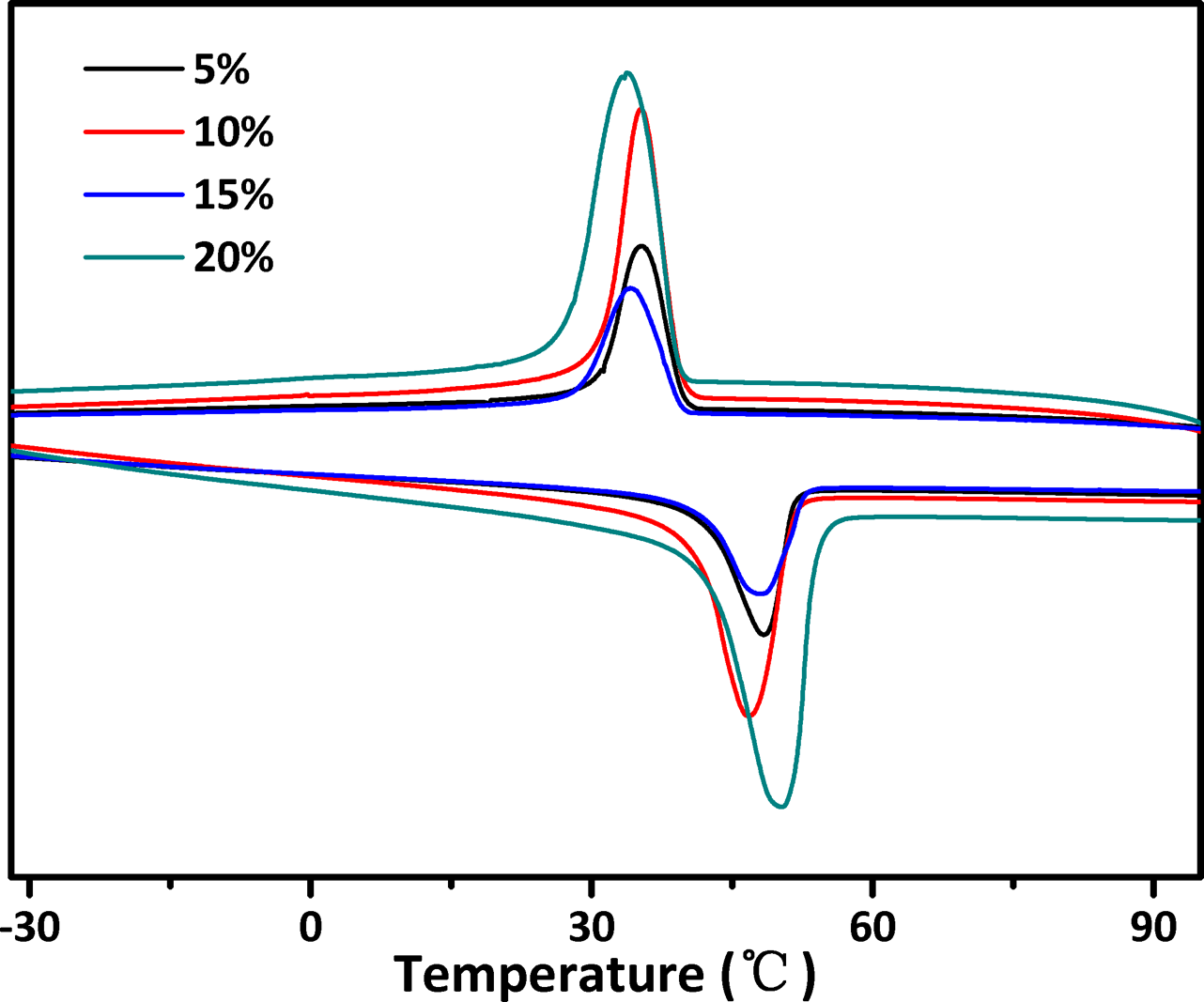


**FIGURE. S8** DSC analyses of tetra-PEG hydrogels.


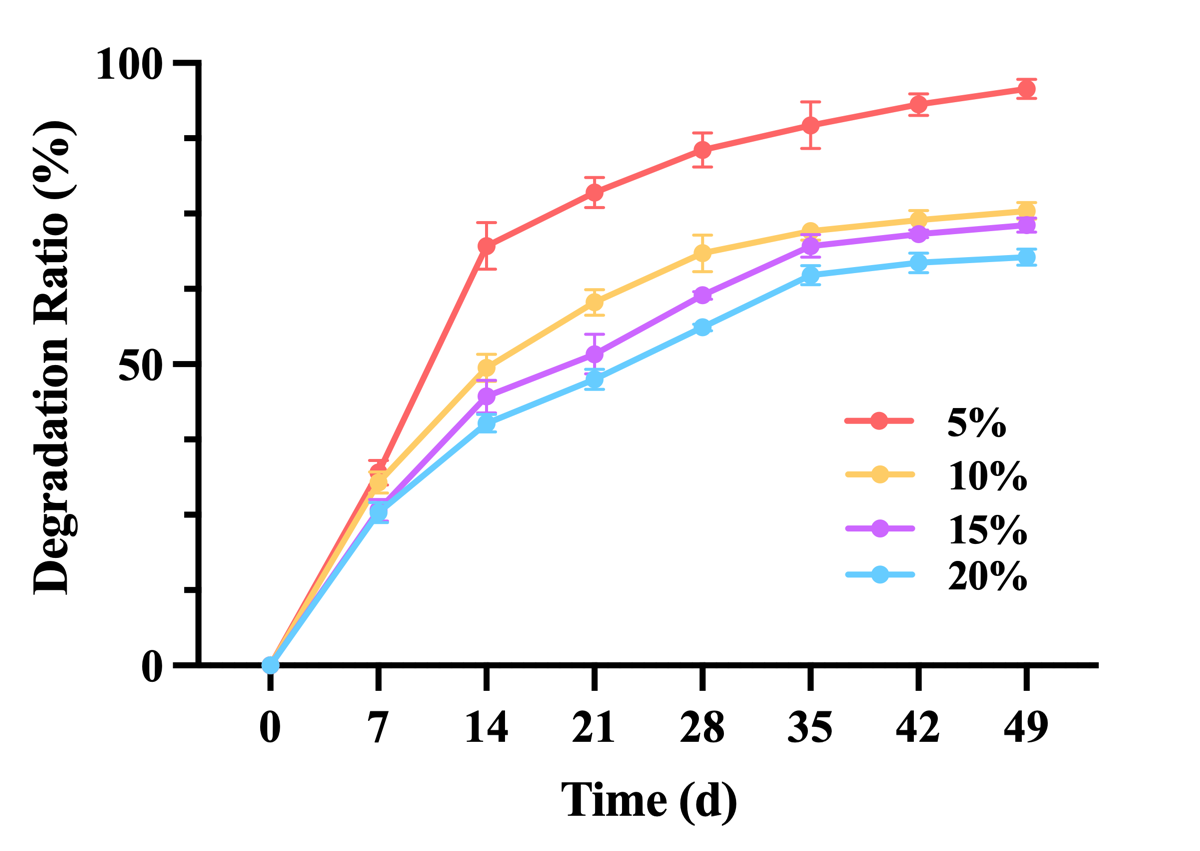


**FIGURE. S9** Enzymatic degradation of tetra-PEG hydrogels.

**FIGURE. S10** Live/dead assay showing the distribution and survival of rabbit chondrocytes within the tetra-PEG hydrogel.
